# The debiasing paradox: cognitive biases in judicial adjudication of valuation adjustment mechanisms across Chinese and American courts

**DOI:** 10.3389/fpsyg.2026.1870936

**Published:** 2026-08-27

**Authors:** Zihui Gao

**Affiliations:** Shandong University of Science and Technology, Qingdao, China

**Keywords:** anchoring effect, archival study, cognitive bias, commercial adjudication, content analysis, debiasing paradox, judicial decision-making, valuation adjustment mechanism

## Abstract

**Background:**

Judicial adjudication of share repurchase clauses in Chinese valuation adjustment mechanisms (VAMs) exhibits marked typological divergence. The Ninth Meeting Minutes, issued in November 2019, sought to unify adjudicatory standards; however, disagreement has persisted. Existing scholarship accounts for this divergence through doctrinal analysis, leaving systematic cognitive distortions in judicial reasoning unexplored.

**Objective:**

This study advances a “debiasing paradox” thesis—that a judicial policy instrument designed to harmonize standards may, through a “normative anchor” mechanism, introduce new systematic cognitive biases. Treating anchoring as the core bias and framing effect and availability heuristic as downstream accompaniments, it examines how all three manifest in Chinese and American VAM adjudication and tests a hierarchical transmission model.

**Methods:**

Judicial reasoning sections from 217 Chinese VAM rulings (China Judgements Online) and 63 American earn-out rulings (Westlaw) underwent double-blind process coding (Cohen’s *κ* = 0.71–0.78). Cross-sectional comparison tested anchoring (post-2019 VAM vs. private lending baseline, *n* = 42); interrupted time series analysis (ITSA) tested changes in framing effect and availability heuristic around the Minutes; configural frequency analysis (CFA) identified bias co-occurrence patterns.

**Results:**

The VAM anchoring rate (47.7%) significantly exceeded that of private lending (26.2%), χ^2^(1) = 5.87, *p* = 0.015, Cramér’s *V* = 0.186. After the Minutes, the framing effect displayed an immediate level shift (*β*₂ = 12.4, *p* = 0.024), while the availability heuristic exhibited a delayed trend increase (*β*₃ = 3.2, *p* = 0.027), a pattern consistent with the hierarchical transmission hypothesis. CFA revealed significant over-representation of the “anchor–frame coupling” pattern (z = 2.63, *p* = 0.009).

**Conclusion:**

The findings provide preliminary archival evidence consistent with the Minutes having produced indirect biasing effects through the hierarchical transmission of a normative anchor—triggering framing downstream and reinforcing availability indirectly—pointing to a debiasing paradox inherent in institutional tools for reducing adjudicatory divergence.

## 1 Introduction

### 1.1 Adjudicatory divergence over VAM repurchase clauses

Functionally identical share repurchase clauses embedded in valuation adjustment mechanisms have received diametrically opposite judicial treatment across Chinese courts. One court characterizes a repurchase clause triggered by missed performance benchmarks as a negotiated investment risk-allocation arrangement and upholds it; another court treats an equivalent clause as a disguised fixed-return arrangement and invalidates it on the grounds that it violates the capital maintenance principle under company law. This divergence is not confined to courts at different hierarchical levels—it also surfaces among courts of comparable jurisdiction adjudicating disputes involving substantially identical contractual structures. Lin (2020) documented this pattern systematically through empirical analysis of hand-collected judgments from China Judgements Online.

The institutional trajectory of VAM adjudication in China has been marked by abrupt directional shifts rather than incremental doctrinal consolidation. The Supreme People’s Court’s guiding stance oscillated between permissive and restrictive orientations before it issued, in November 2019, the Ninth Meeting Minutes—a comprehensive policy document intended to provide a definitive analytical framework for VAM disputes. The Minutes were explicitly conceived as a unifying instrument, designed to eliminate adjudicatory inconsistencies that had undermined investment certainty and inflated transaction costs. Wang (2022), through empirical analysis of post-Minutes rulings, demonstrated that divergence persists in altered form: courts no longer disagree over whether to apply the Minutes’ analytical framework, but over how to apply it to specific factual situations. This persistence raises a question that doctrinal analysis cannot fully address. If divergence survives a comprehensive guidance document specifically engineered to eliminate it, the source of inconsistency may not reside entirely in the ambiguity of legal rules but may also reflect deeper cognitive mechanisms operating within the adjudicatory process itself.

### 1.2 Limitations of existing research and the cognitive psychology perspective

The dominant scholarly approach to VAM adjudicatory divergence has been doctrinal analysis—examining whether courts correctly interpreted contractual terms, properly applied statutory provisions, and faithfully followed the analytical pathway prescribed by the Minutes. This approach remains indispensable for identifying errors in legal reasoning, but it tacitly assumes that adjudicatory divergence is primarily an interpretive disagreement about the content of legal rules. An alternative explanatory framework rooted in cognitive psychology suggests that systematic patterns of divergence may reflect not disagreement about what the law requires, but predictable distortions in the cognitive processes through which judges arrive at their conclusions.

This alternative framework rests on substantial empirical foundations. Rachlinski and Wistrich (2017) synthesized over two decades of controlled experiments involving sitting judges and concluded that judicial decision-making exhibits systematic reliance on intuitive heuristics—including anchoring, framing, and availability—across civil, criminal, and administrative contexts. Guthrie et al. (2001) provided early experimental evidence that federal magistrate judges are susceptible to the same cognitive biases as the general population, challenging the assumption that judicial reasoning is immune to heuristic influence. Spamann and Klöhn (2016) experimentally demonstrated that legally irrelevant factors accounted for a considerable share of variance in federal judges’ decisions. Olaborede and Meintjes-Van der Walt (2020) confirmed the prevalence of cognitive biases across hearing, adjudication, and sentencing stages.

Two methodological gaps in this literature constrain its applicability to the VAM context. Berthet (2022), in a review of cognitive biases among professional decision-makers across four occupational domains, identified an over-reliance on vignette-based experimental designs and insufficient attention to individual differences as structural weaknesses. While vignettes afford internal validity, they cannot capture the institutional pressures, time constraints, and doctrinal complexity that may amplify or reduce biases in actual adjudication. Teichman et al. (2023) further specified that cognitive biases prove most resistant to professional training in legal domains characterized by ambiguity and weak feedback mechanisms—conditions that accurately describe VAM adjudication. VAM disputes occupy a doctrinal intersection where contract law, company law, and investment regulation converge without clear hierarchical ordering; judges must navigate competing legal frameworks without the kind of stable, well-rehearsed analytical routine that might enable a reliable System 2 override of heuristic intrusions. From the Chinese context specifically, Liu and Li (2019) provided direct evidence, experimentally demonstrating that Chinese judges deploy doctrinal reasoning techniques to rationalize decisions actually driven by cognitive biases, rendering bias invisible in written judgments while the underlying cognitive distortion remains intact. This finding carries methodological implications for the present study: if bias traces can be concealed through doctrinal rationalization, the process coding method employed here may underestimate true bias prevalence—a limitation that is acknowledged but does not invalidate the detection of systematic patterns across large samples.

A critical precedent exists in the criminal sentencing literature. Bennett (2014), writing as a sitting U.S. federal judge, documented how the Federal Sentencing Guidelines—designed to promote uniformity—simultaneously functioned as cognitive anchors, compressing sentencing distributions toward the guideline range and introducing biases that did not exist prior to the guidelines’ adoption. This finding has never been extended from criminal sentencing to commercial adjudication, where policy instruments operate through qualitative analytical frameworks rather than numerical ranges. Liu et al. (2021) documented bias susceptibility in Chinese commercial adjudication but did not examine whether institutional instruments intended to reduce inconsistency might themselves become sources of bias.

### 1.3 Research questions and contributions

This study poses three research questions bridging doctrinal analysis of VAM divergence with the cognitive psychology of judicial decision-making.

RQ1: Under the guidance of the same policy instrument, does the degree of legal standard ambiguity amplify the anchoring effect of a normative anchor?

RQ2: Does a normative anchor produce indirect biasing effects through a hierarchical transmission mechanism—triggering framing effects downstream and indirectly reinforcing the availability heuristic?

RQ3 (Exploratory): Do cognitive bias patterns in VAM/earn-out adjudication across Chinese and American jurisdictions differ in a manner consistent with the presence or absence of an institutional anchor?

**Figure 1:**
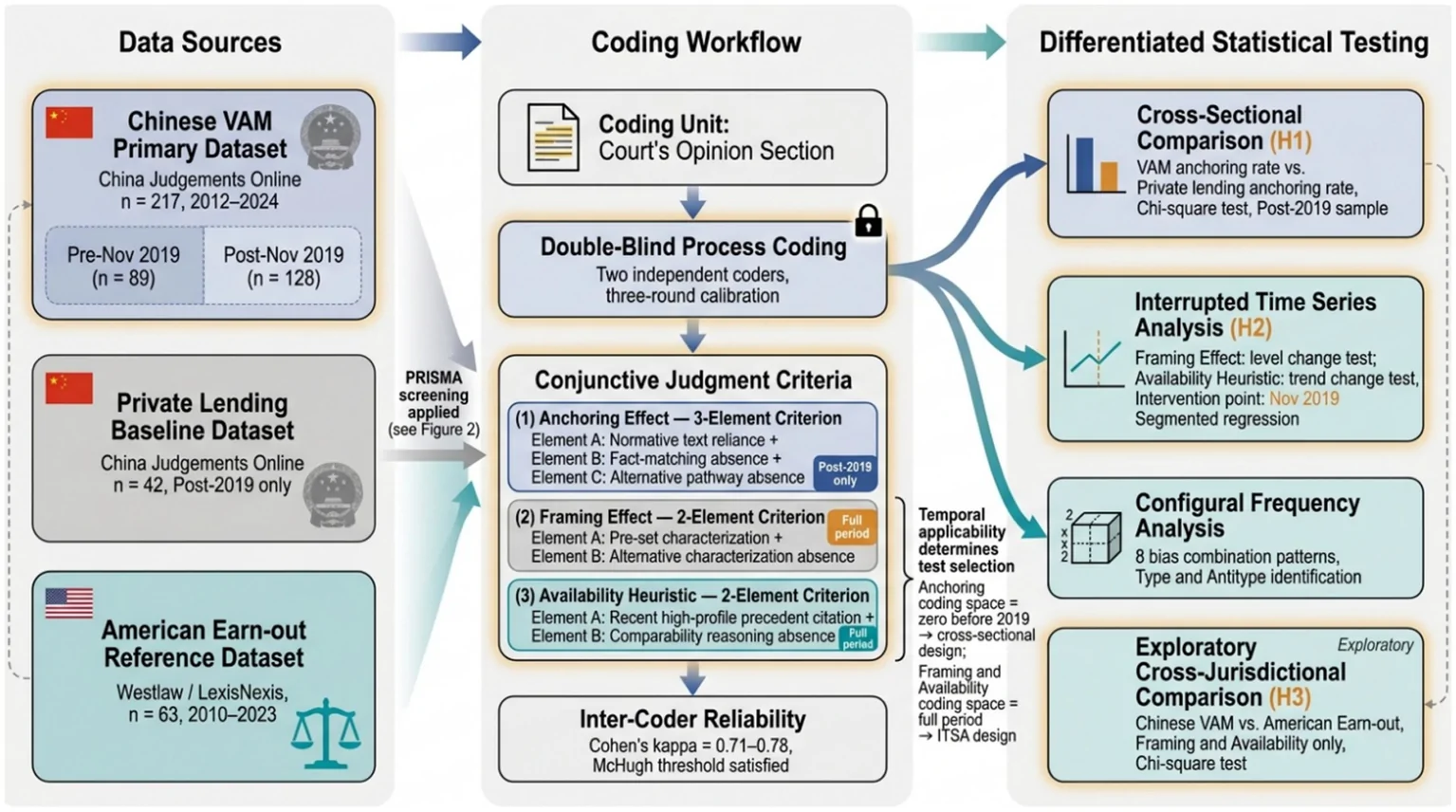
Overall research design framework, visualizing the relationships among the three datasets (Chinese VAM primary dataset, private lending baseline, and American earn-out reference), the double-blind process coding workflow with conjunctive judgment criteria for each bias, and the differentiated statistical testing logic: Cross-sectional comparison for anchoring effect (*H1*), interrupted time series analysis for framing effect and availability heuristic (*H2*), configural frequency analysis for bias co-occurrence patterns, and exploratory cross-jurisdictional comparison (*H3*). The temporal applicability of each bias’s coding criteria determines the matched analytical strategy.

The study offers three interrelated contributions. At the theoretical level, it advances the “debiasing paradox” thesis along with the concept of a “normative anchor” and its hierarchical transmission mechanism, extending Bennett’s (2014) findings from criminal sentencing into commercial adjudication and shifting the analytical focus from “whether an anchor exists” to “how an anchor transmits biasing effects through downstream cognitive mechanisms.” The anchoring typology is thereby expanded from irrelevant anchors (Englich et al., 2006), relevant anchors (Enough and Mussweiler, 2001), and numerical institutional anchors (Bennett, 2014) to include qualitative institutional anchors that have downstream transmission capacity. At the methodological level, responding to Berthet’s (2022) call for greater ecological validity, the study introduces “process coding” of judicial reasoning texts—moving beyond outcome-level analysis to identify cognitive bias indicators within the argumentative structure of judicial reasoning. At the empirical level, it provides evidence of how the institutional environment shapes the reasoning patterns associated with cognitive biases in commercial adjudication, employing a combined cross-sectional comparison and interrupted time series design that disentangles direct anchoring effects from indirect transmission effects. Lin (2020) established the empirical foundation by documenting VAM adjudicatory divergence; this study identifies cognitive mechanisms that explain why divergence persists even after a unifying policy instrument was introduced. The overall research design framework is presented in Figure 1.

The remainder proceeds as follows. The next section presents the theoretical framework, followed by institutional backgrounds, methodology, results, discussion, and conclusion.

## 2 Theoretical framework

### 2.1 Dual-process theory and the vulnerability of VAM adjudication

Dual-process theory distinguishes two modes of cognitive processing: System 1 operates automatically, rapidly, and with minimal effort, while System 2 engages in controlled, effortful, rule-driven reasoning (Kahneman, 2011). Judicial decision-making has long been presumed to exemplify System 2 processing—trained professionals applying legal rules through structured deliberation. However, empirical evidence challenges this presumption. Rachlinski and Wistrich (2017), synthesizing experiments involving hundreds of sitting judges, found that judges as a group scored on the Cognitive Reflection Test in a pattern consistent with intuitive rather than reflective cognitive styles and that their decisions were systematically influenced by anchoring, framing, and other heuristics across multiple legal contexts. Guthrie et al. (2007) formalized this as an “intuitive-override” model of judicial decision-making, proposing that judges’ decisions typically originate in rapid System 1 assessments that are subsequently rationalized through System 2 legal reasoning, rather than being genuinely produced by deliberate analysis from the outset.

Judicial susceptibility to heuristic bias is not uniform across legal domains. Kahneman and Klein (2009) identified three conditions under which professional intuition can achieve accuracy: the decision environment must present stable regularities, outcomes must be sufficiently predictable to support pattern recognition, and the professional must receive timely feedback on prior decisions. VAM adjudication satisfies none of these conditions. As a relatively novel contractual form in Chinese commercial law, VAM disputes lack accumulated precedent that generates stable environmental regularities. Applicable legal standards have undergone rapid, discontinuous evolution—from the Supreme People’s Court’s 2012 Haifu ruling, through the 2019 Huagong ruling, to the Ninth Meeting Minutes—depriving judges of the predictable landscape needed to calibrate their intuition. Teichman et al. (2023) supplied converging evidence: legal professionals in their experimental sample resisted cognitive biases in domains with established reference frames but showed no more resistance than laypeople in domains characterized by ambiguity and normative flux. VAM adjudication, situated at the intersection of contract law, company law, and investment regulation, embodies the kind of multi-framework ambiguity in which professional training offers the least protection against heuristic intrusion.

### 2.2 Hierarchical transmission: normative anchors and downstream effects

The anchoring effect—the tendency to assimilate judgments toward an initially encountered value under uncertainty—was established by Tversky and Kahneman (1974) as a pervasive feature of human judgment. Within judicial contexts, the concept has undergone progressive typological refinement. Enough and Mussweiler (2001) found that prosecutors’ sentencing recommendations act as “relevant anchors”: even when experienced judges consciously dismiss such recommendations as adversarial positions, sentencing decisions are systematically pulled toward the recommended value. Englich et al. (2006) demonstrated that even numbers entirely irrelevant to the adjudicatory task—random figures from a dice roll—significantly displaced judges’ sentencing judgments, revealing the automaticity of anchoring. Bennett (2014) extended this to the institutional level, documenting how the Federal Sentencing Guidelines operated as a “numerical institutional anchor,” compressing outcomes toward guideline midpoints regardless of case-specific facts. Bystranowski et al. (2021) provided quantitative corroboration through a meta-analysis of 45 legal decision-making experiments, confirming that relevant anchors reliably produce larger effect sizes than irrelevant ones. Glöckner and Englich (2015) further specified this relationship: anchors perceived by decision-makers as legitimate and pertinent generate disproportionately stronger assimilation effects—a finding with direct implications for policy documents carrying doctrinal authority.

The typological progression from irrelevant to relevant to institutional anchors reveals a pattern: as the anchor’s perceived legitimacy and task relevance increase, so does the magnitude of the anchoring effect. Irrelevant anchors (dice rolls) produce moderate displacement despite being transparently unconnected to the judgment task; relevant anchors (prosecutorial recommendations) produce larger displacement because they carry informational content that judges cannot entirely disregard; numerical institutional anchors (sentencing guideline ranges) produce the strongest documented effects because they combine informational relevance with institutional authority—judges who deviate from the guideline range bear the burden of justification, creating an asymmetric incentive structure that reinforces cognitive anchoring through institutional pressure. This escalation pattern generates a theoretical prediction: an anchor that carries both institutional authority and qualitative analytical content—prescribing not merely a number but an entire reasoning pathway—should produce anchoring effects at least as strong as numerical institutional anchors while simultaneously influencing dimensions of judgment that numerical anchors leave untouched.

Building on this progression, the present study proposes the concept of a “normative anchor”—a judicial policy instrument supplying not a specific numerical reference point but a prescriptive analytical pathway. Article 5 of the Ninth Meeting Minutes established a three-stage review sequence for VAM share repurchase clauses—contract validity review, performance condition review, and judicial adjustment—channeling judicial reasoning into a determinate analytical structure. The critical distinction between normative and numerical anchors lies in their scope of influence: numerical anchors displace only quantitative judgments (sentence length, damage amounts), whereas normative anchors simultaneously constrain qualitative determinations (the legal characterization of a transaction) and quantitative outcomes. This dual influence endows normative anchors with a capacity for downstream bias transmission that numerical anchors lack. The downstream transmission capacity can be specified more precisely. A numerical anchor operates through a single cognitive channel: it displaces the quantitative estimate toward the anchor value, but the decision-maker’s qualitative framing and information retrieval strategy remain unaffected. A normative anchor operates through three channels simultaneously. First, it fixes the analytical starting point (anchoring proper). Second, the prescribed pathway carries an embedded qualitative frame that activates a particular interpretive lens—channeling the decision-maker into one characterization of the problem rather than another (framing transmission). Third, by generating a body of anchor-consistent outputs that become publicly available, the normative anchor progressively reshapes the information environment from which future decision-makers retrieve reference material (availability transmission). These three channels correspond to the three biases examined in this study and predict distinct temporal signatures: anchoring and framing should manifest simultaneously upon the anchor’s introduction, while availability reinforcement should emerge with a delay proportional to the time required for anchor-consistent outputs to accumulate.

The normative anchor construct must be distinguished from three neighboring concepts with which it could be conflated. First, it differs from legal formalism. Formalism denotes a normative commitment to rule-bound adjudication and a corresponding style of justification; a formalist opinion may nonetheless engage in extensive, case-specific adjustments and remain open to competing characterizations. The normative anchor, by contrast, is a claim about a cognitive mechanism whose defining feature is insufficient adjustment away from a prescribed starting point (Tversky and Kahneman, 1974). Formalistic reasoning is thus neither necessary nor sufficient for normative anchoring: an opinion can be formalist yet adequately adjusted, or informal in style yet anchored. Second, it differs from precedent effects. Precedent operates through the doctrinal authority of prior individual cases; the normative anchor operates through the analytical pathway prescribed by a single authoritative guidance document, and its distinctive property is a downstream transmission capacity—the simultaneous activation of an embedded qualitative frame and the delayed reshaping of the retrieval environment—that the precedent effects literature does not theorize. Availability transmission, examined separately below, is precisely the channel through which precedent-like effects arise as a downstream consequence of, rather than a substitute for, the anchor. Third, it differs from institutional conformity. Conformity describes strategic or social compliance—following a prescribed pathway to avoid reversal on appeal or peer criticism—and operates at the level of incentives rather than cognition. The two are analytically separable: conformity predicts adherence to the pathway regardless of whether adjustment is adequate, whereas normative anchoring predicts adherence coupled with specific textual signatures of insufficient adjustment (absent fact-matching and suppressed alternatives). The institutional incentives characteristic of the Chinese judicial environment—examined in the Institutional Background—may reinforce anchoring, but the coding criteria are constructed to register the cognitive signature rather than conformity as such.

A potential objection must be addressed at the outset: reliance on the analytical structure prescribed by an authoritative guidance document may reflect legitimate legal reasoning rather than cognitive anchoring. This study draws a principled distinction between the two. Anchoring, in the sense established by Tversky and Kahneman (1974), denotes not the use of a reference point but insufficient adjustment away from it once case-specific information is encountered. Legitimate structured reasoning and cognitive anchoring therefore share an identical starting point—the prescribed pathway of the Minutes—but diverge in what follows: legitimate reasoning adjusts the initial framework to the specific factual configuration of the case and remains open to alternative characterizations, whereas anchoring is marked by insufficient adjustment and the suppression of alternative starting points. Critically, the institutional legitimacy of the anchor does not immunize reasoning against this distortion. Glöckner and Englich (2015) demonstrated that anchors perceived as legitimate and task-relevant generate disproportionately stronger assimilation effects than irrelevant ones; thus, the authority of the Minutes does not suggest weaker anchoring but rather stronger anchoring—provided that adjustment is, in fact, insufficient. The empirical question is therefore not whether judges rely on the Minutes (reliance is near-universal and frequently appropriate) but whether that reliance is accompanied by the adjustment that legitimate reasoning requires. This distinction is implemented operationally in the coding framework: citation of the Minutes (Element A) establishes only the presence of a potential anchor, and a bias coding requires its conjunction with absent fact-matching argumentation (Element B) and absent alternative pathways (Element C)—the textual signatures of insufficient adjustment. The criteria accordingly identify reasoning patterns consistent with anchoring rather than directly observed cognitive states, a constraint examined further in the Discussion.

The same conjunctive logic addresses a family of non-cognitive explanations for the coded patterns. Reliance on the Minutes without extended discussion may reflect legitimate doctrinal reasoning, adherence to judicial writing conventions, or the avoidance of unnecessary repetition rather than psychological anchoring. Each of these alternatives can plausibly account for the absence of a single element—an economical drafting style, for instance, may omit an explicit statement of alternative pathways—but is far less able to account for the simultaneous absence of both case-specific fact-matching (Element B) and any alternative analytical framework (Element C) in conjunction with exclusive reliance on the prescribed pathway (Element A). Requiring all three signatures to co-occur raises the evidentiary threshold against mistaking formalistic or abbreviated reasoning for anchoring, though it cannot eliminate the possibility entirely. The criteria are accordingly framed throughout as identifying reasoning patterns consistent with anchoring rather than as directly demonstrating cognitive bias.

Framing effect—the systematic reversal of preferences depending on whether options are presented as gains or losses (Tversky and Kahneman, 1981)—constitutes the first downstream consequence of normative anchoring. The classic paradigm demonstrates framing through within-subject manipulation; however, judicial adjudication does not permit this, as a judge encounters one case framed in a single way. What archival data can capture is frame-dependent judgment—Rachlinski and Wistrich (2018) showed that judges operating within a single frame rarely consider whether an alternative characterization would alter their conclusion, resulting in single-frame lock-in in written reasoning.

In the VAM context, two competing characterizations map onto the gain-loss distinction with functional precision. The company law capital maintenance framework treats repurchase as potential asset extraction prejudicing creditors—a loss frame. The contract law party autonomy framework treats the same clause as freely negotiated consideration—a gain frame. These focus attention on different aspects of the same transaction and generate systematically different conclusions, constituting functionally distinct cognitive frames in Tversky and Kahneman’s (1981) sense. The Minutes’ review sequence—requiring company law assessment before contract law analysis—channels judges into the loss frame at the outset. Once anchored to this pathway, the embedded frame is simultaneously activated through the structural logic of the prescribed sequence—a “downstream triggered” mechanism.

Availability heuristic—the tendency to estimate frequency or probability based on ease of retrieval (Tversky and Kahneman, 1973)—represents a more indirect downstream effect. Liu et al. (2021) provided direct experimental evidence that Chinese judges, despite operating in a system formally disclaiming precedential constraint, are cognitively influenced by prior decisions in a pattern functionally indistinguishable from precedent effects. Following the Minutes, a proliferation of “Minutes-compliant” decisions became publicly available on China Judgements Online, constituting the most readily retrievable cognitive material for judges adjudicating subsequent VAM disputes. The availability heuristic is thus reinforced not directly by the normative anchor itself but indirectly through the accumulation of anchor-consistent precedents that dominate judges’ information retrieval environment. This indirect pathway entails a temporal lag: unlike framing, which is triggered simultaneously with anchoring, availability-driven bias requires a sufficient volume of Minutes-compliant decisions to accumulate before the mechanism is substantially amplified. The three biases therefore form a hierarchical transmission structure—the normative anchor operates as the upstream cause, the framing effect is triggered as an immediate downstream accompaniment, and the availability heuristic is reinforced through an indirect pathway with a predictable delay. System 1 processing (Kahneman, 2011) facilitates automatic propagation across levels, as all three biases operate through associative and retrieval-based mechanisms that bypass deliberative analysis.

### 2.3 The debiasing paradox and research hypotheses

The hierarchical transmission structure outlined above gives rise to a counterintuitive proposition. The “debiasing paradox” refers to the phenomenon whereby a judicial policy instrument designed to reduce inconsistency simultaneously generates new systematic cognitive biases through normative anchoring and its downstream effects. This paradox is not unique to the legal domain. Saposnik et al. (2016) documented a similar pattern in clinical medicine: standardized practice guidelines intended to improve diagnostic consistency instead anchored physicians to guideline-specified parameters, inhibiting individualized assessment. Bennett (2014) identified the same dynamic in U.S. federal sentencing. The paradox reflects a structural tension inherent in any institutional effort to standardize professional judgment—the very features that make a standardizing instrument effective as a coordination tool (salience, specificity, authoritative endorsement) are precisely the features that make it potent as a cognitive anchor.

Empirical testing requires a differentiated strategy because the three biases differ in their temporal applicability across the November 2019 intervention point. The anchoring coding criteria directly reference the Ninth Meeting Minutes, meaning the coding space is zero before 2019. Using ITSA for pre-post comparison would capture only the mechanical expansion of coding eligibility, not genuine cognitive change. The appropriate strategy for anchoring is cross-sectional comparison within post-2019 samples. By contrast, framing effect and availability heuristic coding criteria are operationally independent of the Minutes and have coding space throughout the observation period, enabling meaningful ITSA testing.

#### 2.3.1 H1 (cross-sectional test of anchoring)

In post-2019 samples, anchoring frequency in VAM rulings is significantly higher than in contemporaneous private lending rulings. VAM disputes involve substantially greater legal ambiguity than private lending disputes—the latter governed by mature interest rate caps and abundant precedent—and this ambiguity, under the guidance of the same policy instrument, amplifies susceptibility to normative anchoring. Teichman et al. (2023) demonstrated that legal professionals resist biases in domains with established reference frames but are as vulnerable as laypeople in domains lacking them; Kahneman and Klein’s (2009) boundary conditions for effective professional intuition are systematically absent from VAM adjudication.

#### 2.3.2 H2 (ITSA test of hierarchical transmission)

Following the Minutes, framing effect frequency exhibits a significant level change, reflecting downstream triggering—the normative anchor simultaneously activates the qualitative frame embedded in its pathway. Availability heuristic frequency shows a delayed upward trend, reflecting indirect reinforcement—Minutes-compliant decisions require time to accumulate before dominating judges’ retrieval environment. If both components are supported, the debiasing paradox operates through indirect downstream effects rather than solely through direct anchoring—a finding of greater theoretical depth than the near-tautological observation that “anchoring increases after an anchor is introduced.” Bystranowski et al. (2021) provide the effect size benchmark; Glöckner and Englich (2015) supply the expectation that institutionally legitimated anchors produce effects at the higher end of that range.

#### 2.3.3 H3 (exploratory)

Given the institutional anchor’s presence in the Chinese system and its absence in the American system, anchoring frequency in Chinese VAM rulings is expected to exceed that in American earn-out rulings, with American bias distribution being more dispersed. This hypothesis is designated exploratory due to the interpretive constraints that cross-jurisdictional differences in legal doctrine, judgment-writing conventions, and reasoning transparency impose.

### 2.4 Institutional background

#### 2.4.1 The ninth meeting minutes as normative anchor

Prior to November 2019, Chinese courts adjudicating VAM share repurchase disputes operated amid considerable normative uncertainty. Lin (2020) and Wang (2022) documented that adjudicatory outcomes were not reliably predictable, with conflicting rulings across courts creating a fragmented environment.

Article 5 of the Minutes established a three-stage review pathway: courts were to assess sequentially (i) validity under contract and company law, (ii) whether performance conditions had been met, and (iii) whether judicial adjustment was warranted. This prescriptive sequence constrains not only what judges analyze but also the order and evaluative lens through which they do so—fixing the starting point, direction, and scope of reasoning in a manner functionally analogous to the numerical ranges that Bennett (2014) identified as sentencing anchors, but operating through qualitative analytical guidance rather than quantitative reference points. The three-stage structure simultaneously embeds an anchoring component (fixed analytical steps) and a framing component (by requiring company law assessment before contract law analysis, it implicitly prioritizes capital maintenance), rendering the Minutes a normative anchor with hierarchical transmission capacity.

The Minutes’ cognitive impact is amplified by features of the Chinese judicial institutional environment. Several structural aspects of the Chinese judicial system reinforce judges’ reliance on authoritative guidance documents. Case clearance rate evaluations incentivize swift disposition over independent analysis; higher court guidance opinions, though formally non-binding, carry de facto binding force given that deviation risks reversal on appeal; and mandatory publication of judgments on China Judgements Online generates a peer scrutiny effect that discourages departures from established analytical pathways. Liu et al. (2021) provided experimental evidence that Chinese judges, while formally unconstrained by precedent, exhibit cognitive responses to prior decisions functionally indistinguishable from precedent effects—suggesting the Minutes’ cognitive influence may exceed their formal legal authority.

#### 2.4.2 American earn-outs and the comparative basis

American earn-out clauses serve a function analogous to Chinese VAM clauses—bridging valuation gaps by tying a portion of the consideration to post-closing performance. Disputes are primarily adjudicated through contract interpretation principles and the implied covenant of good faith. The critical institutional distinction is the absence of any centralized guidance document analogous to the Minutes—judges rely on contractual text, case-specific records, and common law precedent, with no single instrument guiding reasoning along a prescribed pathway. Spamann et al. (2021) demonstrated cross-jurisdictionally that common law and civil law judges do not differ systematically in their susceptibility to bias, suggesting that observable differences are attributable to institutional features rather than inherent cognitive differences. The American dataset is therefore positioned as an exploratory contrast rather than a causal benchmark. Four institutional differences limit what the comparison can support. Beyond the differences in legal tradition and judgment-writing conventions already noted, two further asymmetries bear directly on the coded indicators. Judicial hierarchy differs: Chinese VAM disputes are distributed across a four-tier system in which non-binding higher-court guidance exerts de facto influence, whereas American earn-out litigation is concentrated in Delaware and resolved through common law precedent, so the two systems channel reasoning through structurally different authority relations. Publication practices differ: mandatory publication on China Judgements Online yields a near-complete population of reasoned rulings, whereas selective publication and the prevalence of unpublished opinions in the United States mean the American sample is drawn from a more fully reasoned and unrepresentative subset. Each asymmetry can independently raise or depress the textual indicators coded as biases, and none can be neutralized within an archival design; the comparison therefore supports directional inference only.

## 3 Methodology

### 3.1 Research design and methodological positioning

This study employs an archival research design, drawing on publicly available judicial decisions as the primary data source. Within the evidence hierarchy that Berthet (2022) established for cognitive bias research among professional decision-makers, archival studies of actual professional outputs occupy a higher position of ecological validity than vignette experiments—the dominant method in judicial bias research—while accepting a corresponding reduction in internal validity. Rachlinski and Wistrich (2017) noted that the field’s reliance on experimental paradigms has left a gap in understanding how biases manifest amid institutional complexity; the archival approach directly addresses that gap.

The core methodological innovation lies in the shift from “outcome coding”—classifying decisions by result—to “process coding”—identifying bias indicators within the structure of judicial reasoning. Written judgments are *post hoc* rationalizations shaped by argumentation conventions, and the reasoning a judge articulates may diverge from the actual cognitive pathway. This limitation cannot be fully eliminated within the archival paradigm but is manageable within this study’s analytical framework, as discussed in the Institutional Moderation and Methodological Reflections section.

A clarification of the study’s epistemic scope is warranted at the outset. The evidence assembled here directly relates to the textual structure of judicial reasoning—specifically, the presence or absence of fact-matching argumentation, alternative characterizations, and comparability analysis in written opinions—and how that structure varies with the institutional environment in which opinions are produced. Claims about the cognitive processes underlying these textual patterns—anchoring, framing, and availability—are presented as the most parsimonious theoretical account of the patterns, rather than as directly observed states. Judicial opinions are institutional products shaped by writing conventions, hierarchical review, and expectations regarding legal justification, any of which may leave textual traces resembling those coded here; therefore, the inference from text to cognition is indirect and defeasible. What justifies the cognitive interpretation is not any single coding decision but the convergence of three independent strands developed in the Results: the conjunctive coding criteria, which require multiple co-occurring textual signatures before a bias is recorded; the distinct temporal signatures predicted *a priori* and then observed in the interrupted time series; and the configural coupling identified by the configural frequency analysis. The primary contribution of the study is therefore best understood as documenting how an institutional instrument systematically shapes the observable structure of judicial reasoning, with the cognitive mechanism providing the interpretive framework that makes those institutional regularities intelligible.

The study employs a combined cross-sectional comparison and time series analysis design rather than applying a single method across all biases, because the coding criteria for the three biases differ in their temporal applicability relative to the November 2019 intervention point, necessitating matched analytical strategies.

### 3.2 Dataset design and sample selection

#### 3.2.1 Chinese primary dataset

Constructed from China Judgements Online, targeting 150–300 decisions spanning 2012–2024. Search terms combined “valuation adjustment mechanism” with “share repurchase,” “valuation adjustment,” and “buyback clause.” Inclusion criteria: final enforceable rulings; “Court’s Opinion” sections containing no fewer than 200 characters of substantive reasoning. Lin (2020) provided methodological precedent for hand-collecting VAM judgments from this platform.

#### 3.2.2 Private lending baseline dataset

A baseline of 30–50 private lending rulings from the same platform, restricted to post-2019 samples. Private lending is governed by Chapter 5 of the Ninth Meeting Minutes, so judges face a similar choice between independent analysis and reliance on the Minutes’ prescribed pathway. The critical difference: private lending benefits from established legal standards, interest rate caps, and abundant precedent, with legal ambiguity much lower than in VAM adjudication. The baseline sample size of 42 was determined by the availability of post-2019 private lending rulings that simultaneously satisfied two inclusion criteria: (i) explicit invocation of Chapter 5 of the Minutes, ensuring institutional comparability with VAM rulings applying Article 5; and (ii) no fewer than 200 characters of substantive reasoning. Expanding beyond this threshold would have required relaxing one or both criteria, potentially introducing cases where the Minutes were not substantively engaged—undermining the logic of cross-sectional comparison. A parallel constraint governs the primary dataset. In an archival design, sample size is limited by the population of published rulings satisfying the inclusion criteria rather than determined by the researcher: unlike experimental research, where statistical power can be increased by recruiting additional participants, the pool of qualifying decisions on China Judgements Online at the time of data collection sets a ceiling that cannot be raised without either relaxing the inclusion criteria or waiting for further litigation to accumulate. The first option would dilute the comparison logic described above; the second defines a path for future replication rather than a remedy available within the present study. Wang (2022) provides supplementary empirical context.

#### 3.2.3 American reference dataset

A reference dataset of 50–80 earn-out rulings from Westlaw, spanning 2010–2023, positioned for the exploratory comparison in H3, with acknowledged constraints from cross-jurisdictional differences.

### 3.3 Coding framework: conjunctive judgment criteria

#### 3.3.1 Coding unit

The “Court’s Opinion” section of each judgment.

#### 3.3.2 Coding principle

Each bias is operationalized through a set of observable textual features that must be jointly satisfied. When all prescribed elements co-occur, the item is coded as 1; if any element is absent, it is coded as 0. This conjunctive structure minimizes false positives by requiring convergent textual evidence rather than relying on a single indicator. The framework follows Krippendorff’s (2018) content analysis procedures while implementing the process coding innovation described above (Berthet, 2022).

#### 3.3.3 Anchoring effect: three-element conjunctive criterion (applicable only to post-2019 samples)

Element A (authoritative normative text reliance): reasoning directly cites Article 5 of the Minutes by article number or verbatim language. Element B (absent fact-matching argumentation): reasoning does not include a passage systematically comparing case-specific facts against the Minutes’ stipulated conditions. Element C (absent alternative analytical pathway): reasoning considers no legal analytical framework beyond the Minutes’ pathway.

All three satisfied → coded as 1. Coding is implemented only in post-2019 VAM and private lending samples. The theoretical rationale traces to insufficient adjustment from an initial reference point (Tversky and Kahneman, 1974): Element A confirms the presence of an anchor, Element B captures insufficient case-specific adjustment, and Element C captures the suppression of alternative starting points. This conjunctive structure implements the distinction developed in the Theoretical Framework: Element A alone never triggers a bias coding, ensuring that legitimate doctrinal reliance accompanied by adequate case-specific adjustment is systematically excluded from the anchoring category. To illustrate the boundary between anchoring-indicative and legitimate reasoning: a ruling that states, “According to Article 5 of the Minutes, the repurchase clause shall be reviewed in three stages” and then proceeds directly to its conclusion without mapping the case’s specific facts—such as the target company’s actual financial condition, the precise contractual trigger mechanism, or the availability of capital reduction procedures—onto each stage satisfies all three elements and is coded as 1. By contrast, a ruling that invokes the same Article 5 framework but then devotes a substantial paragraph to explaining why the particular facts of the case (e.g., the company had already completed a capital reduction procedure or the repurchase price was tied to audited net asset value rather than a fixed amount) meet or do not meet the Minutes’ stipulated conditions fails Element B and is coded as 0, regardless of whether it considers alternative analytical pathways. This distinction operationalizes the theoretical difference between reliance on an anchor with insufficient adjustment (bias) and reliance on an authoritative source with adequate independent analysis (legitimate reasoning).

#### 3.3.4 Framing effect: two-element conjunctive criterion (applicable to full-period samples)

Element A (pre-set characterization): reasoning, at its argumentative starting point, characterizes the repurchase clause as a specific legal category—such as “in substance a loan,” “constituting an equity transaction,” or “amounting to an investment exit arrangement”— and this characterization functions as the logical starting point of the argument, preceding factual analysis in argumentative structure rather than necessarily in textual sequence. Element B (absent alternative characterization): reasoning does not discuss other possible legal characterizations.

Both satisfied → coded as 1. This operationalization captures frame-dependent judgment rather than within-subject preference reversal: Element A identifies the presence of an initial frame, and Element B identifies the absence of the counterfactual frame necessary to override frame dependence. Together they measure the necessary condition for single-frame lock-in to determine the outcome—a condition Rachlinski and Wistrich (2018) demonstrated reliably produces the decision shifts characteristic of the framing effect (Tversky and Kahneman, 1981). This criterion is operationally independent of the Minutes—before 2019, pre-set characterization might manifest as reliance on the Haifu framework; after 2019, as reliance on the Minutes’ framework. The framing effect thus has valid coding space throughout the observation period, enabling ITSA.

#### 3.3.5 Availability heuristic: two-element conjunctive criterion (applicable to full-period samples)

Element A (recent high-profile precedent citation): reasoning cites a Supreme People’s Court gazette case, guiding case, or case subject to extensive academic commentary published within the preceding 3 years. Element B (absent comparability reasoning): reasoning does not demonstrate the comparability of the cited precedent to the current case on key facts.

Both satisfied → coded as 1. This criterion is similarly independent of the Minutes. Theoretical rationale: Tversky and Kahneman (1973). The three-year recency window for Element A was selected on both theoretical and practical grounds. Theoretically, the availability heuristic is driven by retrieval ease, which decays with temporal distance; precedents published within 3 years are substantially more likely to be cognitively salient than older decisions. Practically, the three-year window captures the period during which post-Minutes decisions would have entered the public database and become searchable—permitting the delayed accumulation mechanism predicted by H2 to manifest within the observation period. To illustrate: a 2021 ruling citing the Supreme People’s Court’s 2019 Huagong decision without analyzing whether the cited case’s factual configuration (involving a listed company subsidiary with specific capital structure constraints) is comparable to the current dispute’s facts (involving, for instance, a wholly private company with different capital adequacy conditions) satisfies both elements and is coded as 1. A ruling citing the same Huagong decision but explicitly identifying three factual parallels and two distinguishing features between the cases fails Element B and is coded as 0.

#### 3.3.6 Control variables

Case dispute amount (log-transformed), court level, adjudication year, word count of the “Court’s Opinion” section, and adjudicatory outcome (plaintiff wins / defendant wins / partial support) (Table 1).

**Table 1:** Coding manual overview: Operationalization of three cognitive biases.

Bias	Element	Operationalization	Conjunctive rule	Applicable period	Bias indicator example	Legitimate reasoning example
Anchoring effect	A — Normative text reliance	Reasoning directly cites Article 5 of the Minutes by number or verbatim language	All three elements must co-occur → coded as 1	Post-2019 only	“According to Article 5 of the Minutes, the repurchase clause shall be reviewed in three stages…” (no further case-specific analysis follows)	“Article 5 of the Minutes provides the general framework; however, the specific facts of this case require the following adjustments…”
	B — Absent fact-matching	Reasoning lacks a passage systematically comparing case facts against the Minutes’ conditions			Court cites the three-stage pathway and proceeds directly to conclusion without mapping facts onto each stage	Court explicitly compares each factual element against the Minutes’ conditions
	C — Absent alternative pathway	Reasoning does not consider any framework beyond the Minutes’ pathway			No discussion of contract law party autonomy, good faith, or other doctrinal frameworks	Court considers both the Minutes’ pathway and a contract law analysis before selecting one
Framing effect	A — Pre-set characterization	Reasoning characterizes the clause as a specific legal category at the argumentative starting point, before detailed factual analysis	Both elements must co-occur → coded as 1	Full period (2012–2024)	“The repurchase clause in this case is in substance a fixed-return lending arrangement” (stated before any factual discussion)	Court first analyzes economic substance through factual examination, then arrives at a characterization
	B — Absent alternative characterization	Reasoning does not discuss other possible legal characterizations			Only the “disguised loan” characterization is discussed	Court discusses both “disguised loan” and “negotiated investment exit” and explains preference
Availability heuristic	A — Recent high-profile precedent citation	Reasoning cites a SPC gazette case, guiding case, or widely discussed case published within the preceding three years	Both elements must co-occur → coded as 1	Full period (2012–2024)	“As established in the recent Huagong case…” (cited without further factual similarity analysis)	“The Huagong case addressed a similar scenario involving [specific comparable facts]…”
	B — Absent comparability reasoning	Reasoning does not demonstrate comparability of the cited precedent on key facts			Court invokes a precedent’s conclusion directly without discussing factual comparability	Court identifies specific parallels and distinguishing features between the precedent and current case

### 3.4 Coding procedure and reliability assessment

All coding was completed by two independent coders trained in Chinese commercial law. Both coders received standardized training on cognitive bias theory prior to formal coding, covering the operational definitions of anchoring, framing, and availability heuristic and their manifestations in judicial reasoning contexts. In the pre-coding calibration phase, the two coders independently coded a pilot sample of 20–30 rulings, then compared results and iteratively revised the coding manual until inter-coder agreement for each bias reached Cohen’s *κ* ≥ 0.70—the “substantial agreement” threshold (McHugh, 2012). Calibration focused especially on boundary cases at the interface between legitimate doctrinal reliance on the Minutes and reliance indicative of anchoring. For the American earn-out dataset, both coders received supplementary training on U.S. earn-out contractual structures and Delaware court reasoning conventions before commencing coding. Following calibration, formal coding was conducted under double-blind conditions: neither coder had access to the other’s coding decisions during formal coding.

Reliability is reported separately for each bias, as the cognitive complexity of coding decisions differs across bias types. Disagreements were resolved through adjudication by a third researcher with expertise in both cognitive psychology and Chinese commercial law, or through structured discussion following Krippendorff’s (2018) recommendations for reconciling coding disagreements. Both initial agreement rates and final post-reconciliation coding are reported, ensuring transparency regarding the scope and nature of disagreements.

### 3.5 Statistical analysis plan

#### 3.5.1 Level 1: descriptive statistics

Coding frequencies and distributions for each bias across datasets. Basic distribution of adjudicatory outcomes for each dataset.

#### 3.5.2 Level 2: cross-sectional comparison of anchoring (H1)

Within post-2019 samples, a chi-square test (or Fisher’s exact test when expected frequencies fall below 5) compares anchoring frequencies between VAM and contemporaneous private lending rulings. Effect size: Cramér’s V.

#### 3.5.3 Level 3: ITSA for framing and availability (H2)

With November 2019 as the intervention point, segmented regression is applied separately to annual coding frequency percentages for framing and availability, following the framework established by Bernal et al. (2017) and implemented using Stata’s ITSA command (Linden, 2015). Post-estimation diagnostics include counterfactual trend projection and effect size measures (Linden, 2017). The aggregation unit is annual; semi-annual aggregation was considered but rejected due to insufficient per-period observation counts for stable estimation. A methodological caveat: with 13 annual observation windows (7 pre-intervention, 6 post-intervention), the segmented regression estimates are subject to the instability inherent in small-sample time series models. Results should be interpreted as preliminary archival evidence rather than definitive causal estimates; replication with larger datasets spanning a longer post-intervention period would strengthen confidence.

#### 3.5.4 Level 4: configural frequency analysis

Based on binary codings for the three biases, CFA (von Eye and Wiedermann, 2022) identifies which of eight possible combination patterns occur significantly more or less frequently than expected under independence. Extended CFA techniques (von Eye et al., 2010) are implemented as sensitivity checks.

#### 3.5.5 Level 5: exploratory cross-jurisdictional comparison (H3)

Chi-square tests compare bias distributions between Chinese VAM and American earn-out rulings. The reasoning section word count is included as a control variable. Results are designated as exploratory.

#### 3.5.6 Robustness checks

Core findings are re-estimated after excluding Supreme People’s Court rulings. Power analyses are reported. Cross-validation is conducted with Lin’s (2020) dataset on temporally overlapping cases (Table 2).

**Table 2:** Descriptive statistics summary for three datasets.

Variable	Chinese VAM primary dataset	Private lending baseline	American earn-out reference
Total cases	217	42	63
Time span	2012–2024	Post-2019 only	2010–2023
Pre-November 2019	89 (41.0%)	—	—
Post-November 2019	128 (59.0%)	42 (100%)	—
Basic-level court	31 (14.3%)	—	—
Intermediate court	113 (52.1%)	—	—
Higher court	62 (28.6%)	—	—
Supreme People’s Court	11 (5.1%)	—	—
Median dispute amount	RMB 26.8 million	—	—
IQR	RMB 8.5 M–72 M	—	—
Mean word count (SD)	847 characters (412)	—	2,341 words (1,187)
Full support for investor	85 (39.2%)	—	—
Partial support	74 (34.1%)	—	—
Claim dismissed	58 (26.7%)	—	—
Anchoring effect (post-2019)	61/128 (47.7%)	11/42 (26.2%)	N/A
Framing effect (post-2019)	54/128 (42.2%)	—	17/63 (27.0%)
Availability heuristic (post-2019)	39/128 (30.5%)	—	15/63 (23.8%)
Framing effect (pre-2019)	21/89 (23.6%)	—	—
Availability heuristic (pre-2019)	16/89 (18.0%)	—	—
Anchoring + Framing co-occurrence (post-2019)	43/128 (33.6%)	—	—
All three biases (post-2019)	14/128 (10.9%)	—	—
Anchoring κ	0.78 (84.4% initial agreement)	—	—
Framing κ	0.74 (81.2% initial agreement)	—	—
Availability κ	0.71 (79.5% initial agreement)	—	—

“—” indicates data not reported or not applicable. Private lending descriptive breakdowns by court level and dispute amount are not reported separately due to limited sample size.

## 4 Results

### 4.1 Coding reliability and bias frequency distribution

**Figure 2:**
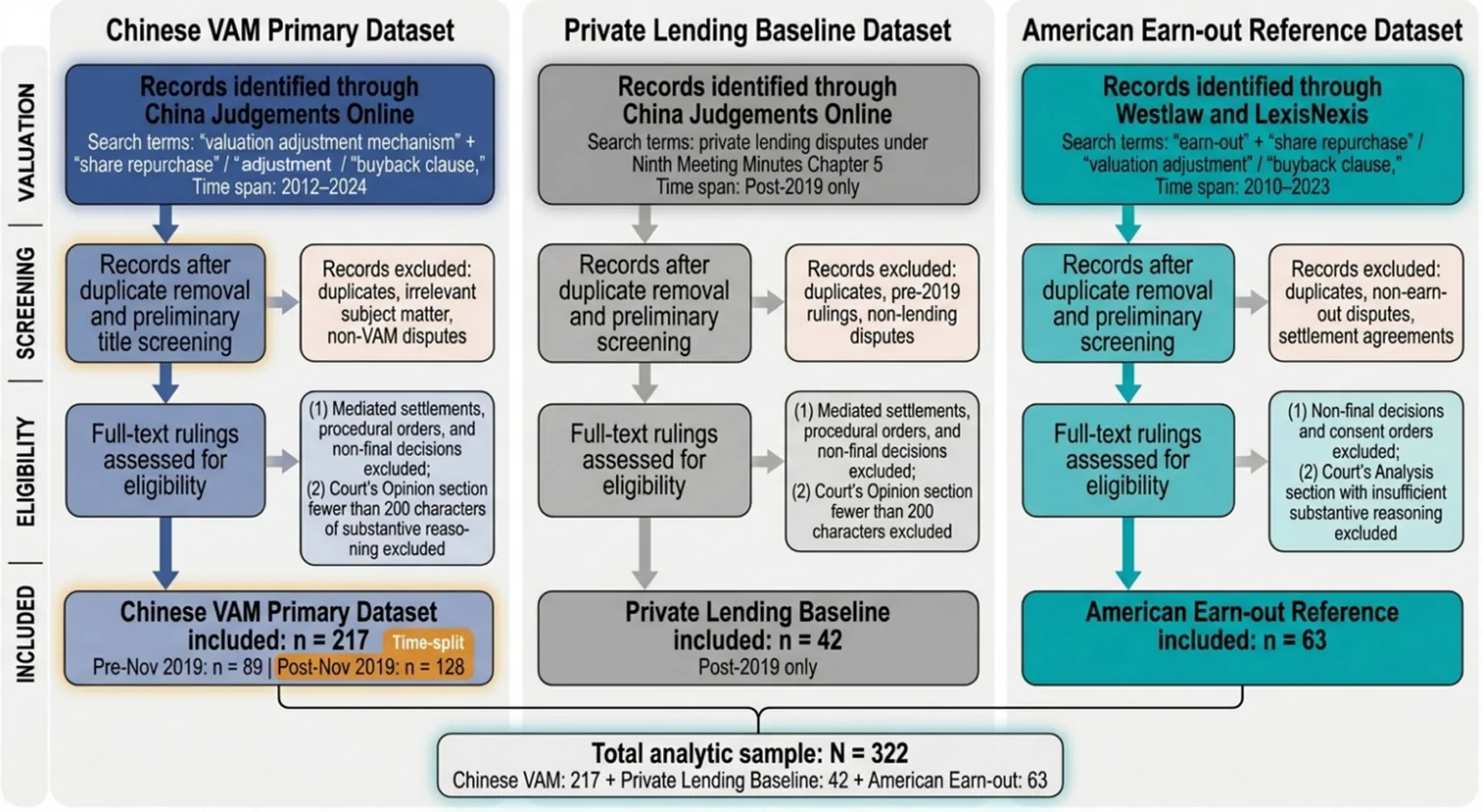
PRISMA flow diagram (Page et al., 2021) documenting the retrieval, screening, eligibility assessment, and final inclusion process for all three datasets. The Chinese VAM primary dataset yielded 217 rulings (pre-November 2019: *n* = 89; post-November 2019: *n* = 128), the private lending baseline yielded 42 rulings (all post-2019), and the American earn-out reference yielded 63 rulings. Total analytic sample: *N* = 322.

Following retrieval and screening (see Figure 2), the Chinese VAM dataset comprised 217 rulings (2012–2024), of which 89 predated November 2019 and 128 were issued on or after that date. The private lending baseline comprised 42 rulings (all post-2019). The American earn-out reference comprised 63 rulings (2010–2023). Within the Chinese sample, intermediate court rulings accounted for 52.1% (113), higher court for 28.6% (62), basic-level for 14.3% (31), and Supreme People’s Court for 5.1% (11). Median dispute amount: RMB 26.8 million (IQR: 8.5–72 million). Adjudicatory outcomes: full support 39.2% (85), partial support 34.1% (74), dismissed 26.7% (58). Mean “Court’s Opinion” word count: 847 characters (SD = 412).

The pre-coding calibration phase proceeded through three iterative rounds (pilot-coding 25, 25, and 15 rulings); formal coding commenced after all biases achieved substantial agreement. The first calibration round revealed the most significant source of inter-coder disagreement: distinguishing between anchoring-indicative reliance on the Minutes and legitimate doctrinal application. Specifically, rulings that cited Article 5 but included brief, formulaic references to case facts—without systematic item-by-item comparison against the Minutes’ conditions—generated coding discrepancies. The coding manual was revised after round one to specify that Element B (absent fact-matching) requires a dedicated analytical passage addressing case-specific facts, rather than merely incidental mention of factual elements within a predominantly Minutes-driven argument. The second round addressed framing effect boundary cases where the legal characterization appeared in the same paragraph as factual analysis, making temporal sequencing ambiguous; the manual was revised to focus on whether the characterization was logically prior to, rather than strictly textually prior to, the factual analysis. The third round addressed a recurrent boundary in the availability criterion: the distinction between legitimate precedent use and availability-driven reliance. Rulings citing a recent high-profile precedent posed difficulty when the comparability discussion was cursory: an opinion invoking the 2019 Huagong decision and noting only that the case was “similar in nature” occupies a gray zone between the two categories. The manual was revised to specify that Element B (absent comparability reasoning) is satisfied only when the opinion transposes the precedent’s conclusion without identifying any case-specific factual parallel or distinguishing feature; an opinion naming even one concrete parallel and one distinction is coded as legitimate precedent use (0), whereas one that imports the holding with no factual bridge is coded as availability-driven (1). Following these revisions, all three biases achieved convergence. This boundary rule operationalizes the difference between reasoning from a precedent’s demonstrated comparability and retrieving a precedent’s conclusion because it is cognitively salient, and accounts for the marginally lower reliability of the availability criterion (*κ* = 0.71).

Formal coding reliabilities: anchoring κ = 0.78 (initial agreement 84.4%, 170 eligible coded samples), framing κ = 0.74 (initial agreement 81.2%, full 217-case sample), availability κ = 0.71 (initial agreement 79.5%, full sample). All exceeded the 0.70 threshold (McHugh, 2012), though availability’s marginally lower reliability reflects the difficulty in judging “absence of comparability reasoning” in boundary cases. Disagreements were resolved through third-researcher adjudication.

In the post-2019 subsample (*n* = 128): anchoring 47.7% (61), framing 42.2% (54), availability 30.5% (39). In the pre-2019 subsample (*n* = 89; anchoring not applicable): framing 23.6% (21), availability 18.0% (16). Co-occurrence: 33.6% of post-2019 rulings (43) were coded for both anchoring and framing; 10.9% (14) for all three biases.

#### 4.1.1 Cross-sectional comparison of anchoring: VAM vs. private lending (H1)

**Figure 3:**
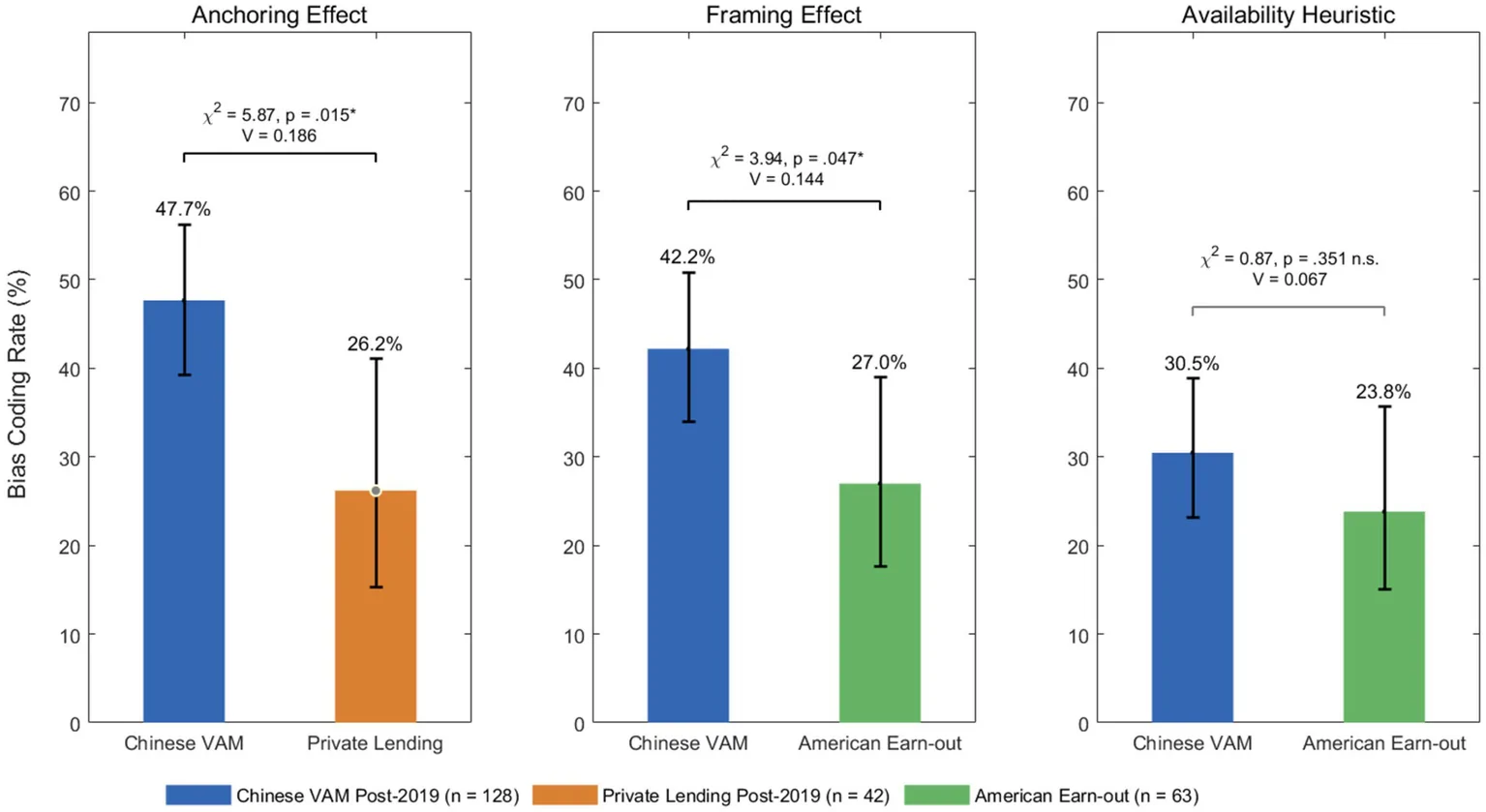
Grouped bar chart of bias coding frequencies for anchoring effect, framing effect, and availability heuristic, grouped by Chinese VAM post-2019 (*n* = 128), private lending baseline post-2019 (*n* = 42), and American earn-out reference (*n* = 63), with 95% confidence interval error bars. The anchoring effect displays only the two post-2019 groups, as coding criteria reference the Ninth Meeting Minutes and are not applicable to U.S. rulings. Statistical significance brackets show chi-square test results and Cramér’s *V* for each pairwise comparison. n.r. = not reported.

In the post-2019 sample, the anchoring coding rate for VAM rulings was 47.7% (61/128), compared with 26.2% (11/42) for private lending rulings (Figure 3). This difference was statistically significant, χ^2^(1) = 5.87, *p* = 0.015, Cramér’s *V* = 0.186. The effect size falls within the small-to-medium range and is comparable to the overall effect in controlled studies reported in Bystranowski et al.’s (2021) meta-analysis.

H1 is supported. Under the same policy instrument, nearly half of VAM rulings exhibited normative anchor capture, whereas only about one-quarter of private lending rulings—governed by more mature standards and richer precedent—did so. Both groups operate under the same Minutes yet show a significant difference, indicating that legal standard ambiguity amplifies anchoring susceptibility even after controlling for the institutional environment. The 26.2% private lending rate is not zero, indicating that the normative anchor retains some influence even in domains with relatively mature standards, though its capture rate is substantially buffered by legal certainty.

#### 4.1.2 ITSA for framing and availability: testing hierarchical transmission (H2)

**Figure 4:**
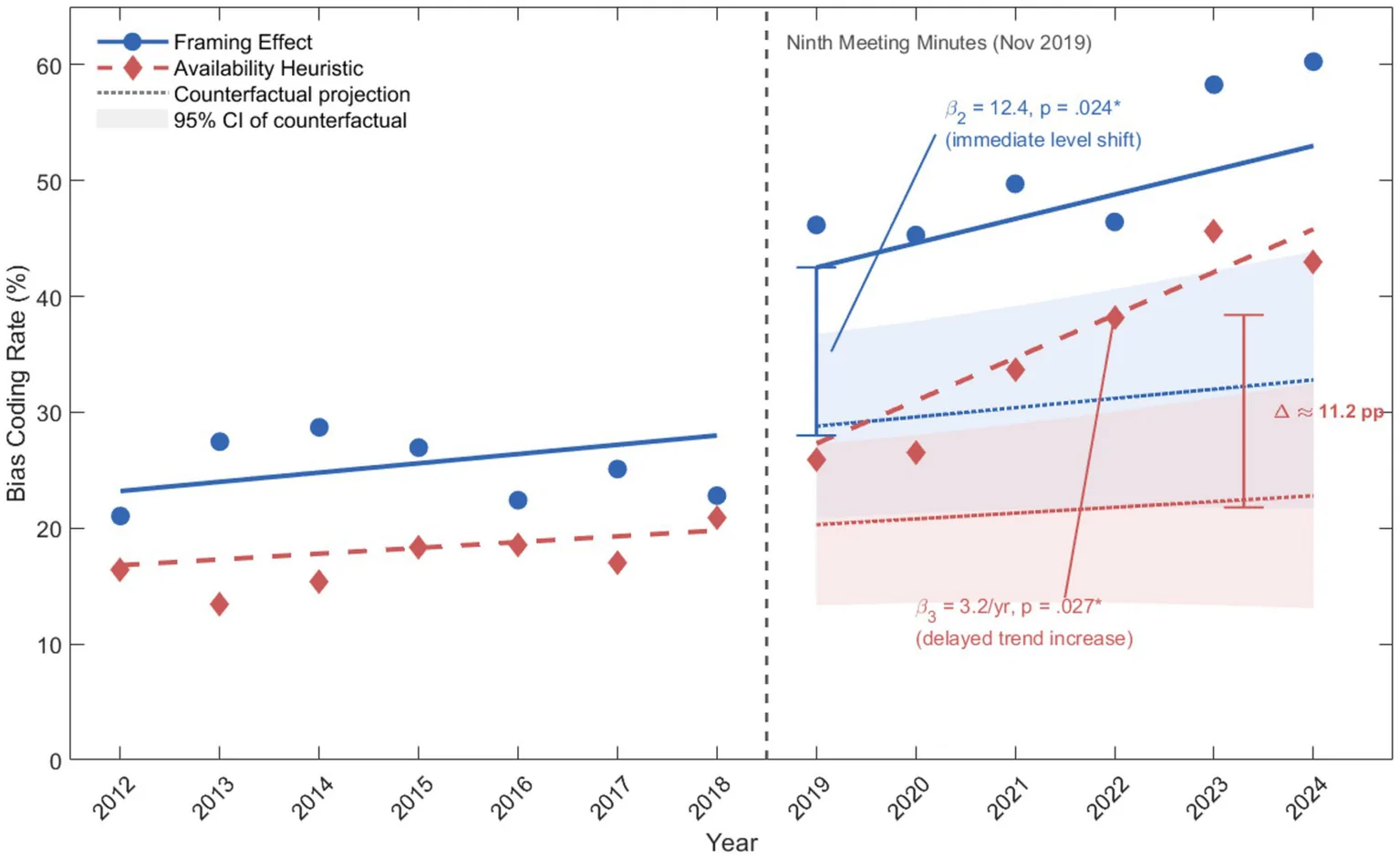
Interrupted time series analysis plots for framing effect (solid line, circle markers) and availability heuristic (dashed line, diamond markers) in the Chinese VAM dataset, 2012–2024. The vertical dashed line marks the November 2019 promulgation of the Ninth Meeting Minutes. Dotted lines represent counterfactual trend projections extending pre-intervention regression slopes into the post-intervention period; shaded bands indicate 95% confidence intervals of the counterfactual projections. The framing effect exhibited a significant immediate level shift ( $\beta_{2}$ = 12.4, *p* = 0.024) with no subsequent trend acceleration ( $\beta_{3}$ = 1.3, *p* = 0.496), consistent with the downstream triggering mechanism. The availability heuristic exhibited no immediate level shift ( $\beta_{2}$ = 3.8, *p* = 0.356) but a significant delayed trend increase ( $\beta_{3}$ = 3.2/yr., *p* = 0.027), consistent with the indirect reinforcement mechanism. By the end of 2022, actual frequency of the availability heuristic exceeded the counterfactual projection by approximately 11.2 percentage points.

Given sample size constraints (217 rulings across 13 annual windows, averaging approximately 17 per window), annual aggregation was adopted. With November 2019 as the intervention point (Figure 4): The interrupted time series yields a temporal association consistent with, but not sufficient on its own to establish, a causal effect of the Minutes; the estimates below are interpreted in that light.

##### 4.1.2.1 Framing effect

Pre-intervention baseline level: approximately 22.4%, with a non-significant pre-intervention trend (*β*₁ = 0.8, SE = 0.61, *p* = 0.228). Level change at the intervention point: *β*₂ = 12.4 percentage points (SE = 4.71, *p* = 0.024), indicating a significant immediate jump. Post-intervention trend change: *β*₃ = 1.3 pp./year (SE = 1.82, *p* = 0.496), non-significant. This pattern—significant level change, non-significant trend change—aligns with the “downstream triggering” mechanism: the normative anchor simultaneously activates the qualitative frame embedded in its pathway, with the effect materializing immediately upon the Minutes’ promulgation rather than accumulating gradually.

##### 4.1.2.2 Availability heuristic

Pre-intervention baseline level: approximately 16.3%, with a non-significant trend (*β*₁ = 0.5, SE = 0.52, *p* = 0.367). Level change: *β*₂ = 3.8 pp. (SE = 3.94, *p* = 0.356), non-significant. Trend change: *β*₃ = 3.2 pp./year (SE = 1.24, *p* = 0.027), significant. This pattern—non-significant level change, significant trend change—matches the “indirect reinforcement” mechanism: availability does not jump immediately but rises progressively as Minutes-compliant precedents accumulate on China Judgements Online. Post-estimation counterfactual analysis (Linden, 2017) showed that by the end of 2022, actual availability frequency exceeded the counterfactual by approximately 11.2 percentage points.

H2 is supported within the limits of the archival design—13 annual observation windows constrain the precision of the segmented regression estimates, as noted in the Statistical Analysis Plan—and the complementary temporal signatures of the two biases merit closer examination. The framing effect’s immediate level shift (*β*₂ = 12.4, p = 0.024) with no subsequent acceleration (*β*₃ = 1.3, *p* = 0.496) suggests a one-time cognitive reconfiguration: once the Minutes established a prescriptive review pathway embedding a particular qualitative frame, judges adjudicating VAM disputes after November 2019 were immediately channeled into that frame, and the framing frequency stabilized at the elevated level without further escalation. This pattern is consistent with downstream triggering, where the normative anchor’s framing component activates at the same moment the anchor itself becomes available—there is no independent accumulation process for framing because the frame is structurally embedded in the anchor’s prescribed pathway rather than constructed from external information.

The availability heuristic’s contrasting pattern—no immediate jump (*β*₂ = 3.8, *p* = 0.356) but a significant progressive acceleration (*β*₃ = 3.2 pp./year, *p* = 0.027)—is equally revealing. The absence of an immediate level shift is less consistent with a direct immediate effect of the Minutes on availability-driven reasoning than with the indirect reinforcement mechanism. Instead, the delayed upward trend is consistent with a gradual accumulation process: Minutes-compliant decisions needed approximately 12–18 months to appear in sufficient volume on China Judgements Online before they began to dominate judges’ precedent retrieval environment. The counterfactual projection showing an 11.2-percentage-point gap by end-2022 indicates that this accumulation process was not only statistically significant but practically meaningful—by the third year after the Minutes, roughly one in ten additional rulings exhibited availability-driven reasoning, a pattern the projection attributes to the anchor’s indirect influence, though the design cannot establish this conclusively. Since neither bias’s coding criteria reference the Minutes, their frequency increases cannot reflect a mechanical expansion of coding eligibility and are most plausibly attributed to indirect cognitive transmission.

### 4.2 Configural frequency analysis

**Figure 5:**
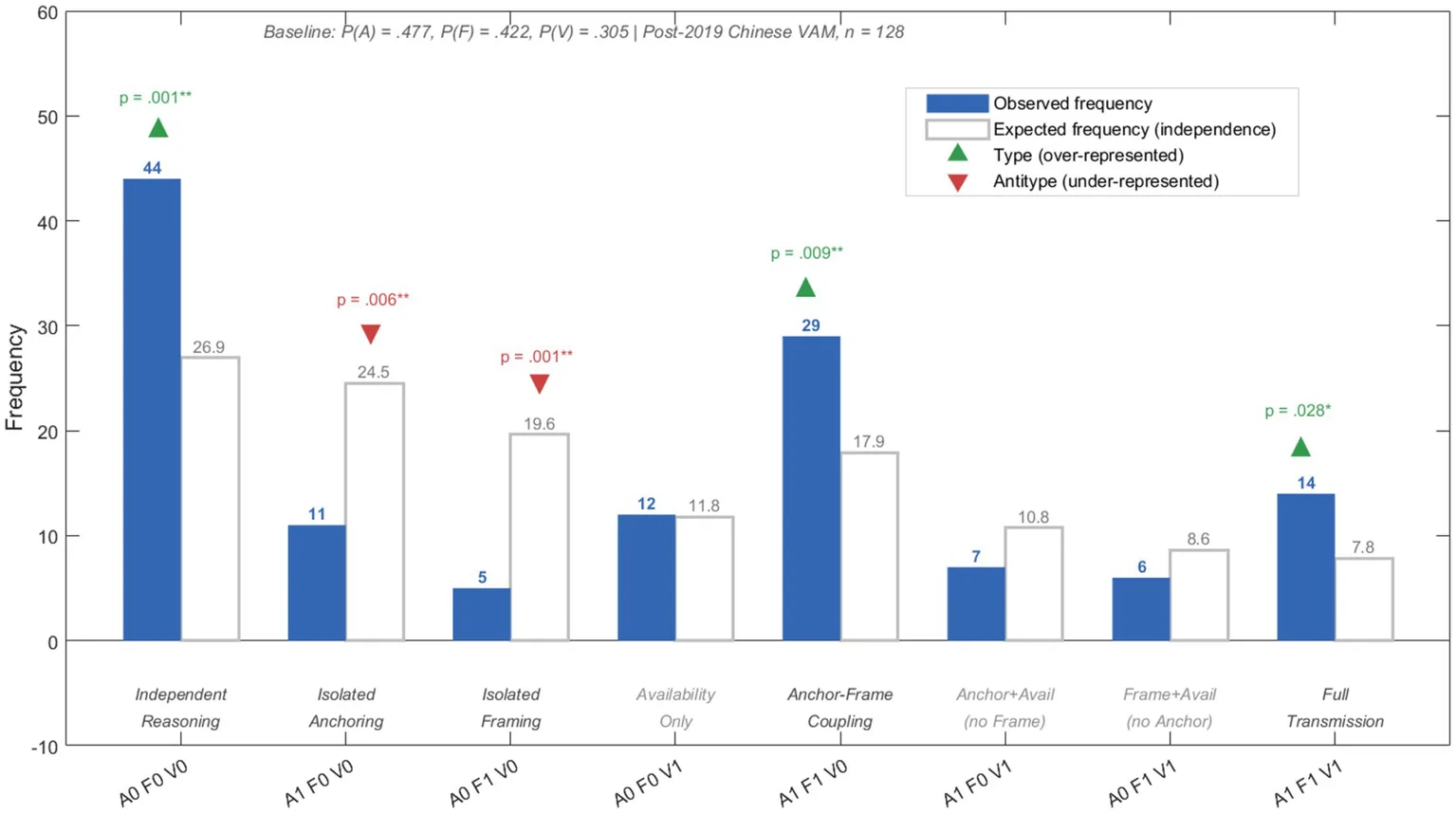
Configural frequency analysis of all eight cognitive bias combination patterns in the post-2019 Chinese VAM subsample (*n* = 128). Solid bars represent observed frequencies; hollow bars represent expected frequencies under the independence model. Green upward arrows (↑) denote significant types (over-represented configurations); red downward arrows (↓) denote significant antitypes (under-represented configurations). Baseline probabilities: *P*(*A*) = 0.477, *P*(*F*) = 0.422, *P*(*V*) = 0.305. The antitype status of “Isolated Anchoring” (*A* = 1, *F* = 0, *V* = 0; observed = 11, expected = 24.52, *z* = −2.73, *p* = 0.006) indicates that anchoring rarely occurs without accompanying framing. The antitype status of “Isolated Framing” (*A* = 0, *F* = 1, *V* = 0; observed = 5, expected = 19.65, *z* = −3.31, *p* = 0.001) similarly indicates that framing rarely occurs without anchoring. Together, these antitypes provide inverse evidence for the structural linkage between anchoring and framing posited by the downstream triggering mechanism. The “Full Hierarchical Transmission” configuration (*A* = 1, *F* = 1, *V* = 1) was over-represented at the individual test level (*z* = 2.20, *p* = 0.028) but reached only marginal significance after Holm correction. *p*-values are Holm-corrected for multiple comparisons. *p < 0.05; **p < 0.01.

Configural frequency analysis tested deviations from expected frequencies under the independence assumption across all eight bias combinations in the post-2019 subsample (*n* = 128; Figure 5). Baseline probabilities: P(A) = 0.477, P(F) = 0.422, P(V) = 0.305.

Three over-represented configurations (types) and two under-represented configurations (antitypes) were identified. The most prominent type was “Anchor–Frame Coupling” (A = 1, F = 1, V = 0): observed 29 (22.7%), expected 17.89, z = 2.63, *p* = 0.009 (Holm-corrected)—lending direct support to the downstream triggering hypothesis. Judges anchored to the Minutes’ pathway simultaneously entered its embedded qualitative frame yet had not been indirectly reinforced by Minutes-compliant precedents at the availability level. “Independent Reasoning” (A = 0, F = 0, V = 0) was likewise a significant type: observed 44 (34.4%), expected 26.93, z = 3.29, *p* = 0.001, indicating that a substantial share of rulings were not captured by any bias coding criterion. “Full Hierarchical Transmission” (A = 1, F = 1, V = 1) yielded an observed frequency of 14 (10.9%) against an expected 7.85, z = 2.20, individual test *p* = 0.028; after Holm correction, however, this result sits at a marginal level and warrants cautious interpretation.

Two antitypes emerged. “Isolated Framing” (A = 0, F = 1, V = 0): observed 5 (3.9%), expected 19.65, z = −3.31, p = 0.001—framing effect in the absence of anchoring was exceedingly rare in the post-2019 subsample. “Isolated Anchoring” (A = 1, F = 0, V = 0): observed 11 (8.6%), expected 24.52, z = −2.73, *p* = 0.006—anchoring seldom appeared without a concurrent framing effect, providing inverse support for the coupling relationship. The remaining three configurations (A = 1, F = 0, V = 1; A = 0, F = 1, V = 1; A = 0, F = 0, V = 1) showed no significant deviations.

The pattern merits interpretation at two levels. At the pairwise level, the robust coupling between anchoring and framing—co-occurrence significantly over-represented at 22.7% versus 14.0% expected, isolated configurations both antitypes—provides the strongest evidence for the downstream triggering hypothesis. If anchoring and framing were independent cognitive events that merely co-occurred in the post-Minutes environment, isolated instances of each should appear at expected frequencies. Their systematic under-representation indicates a structural linkage: when anchoring occurs, framing is triggered as a downstream accompaniment in a proportion substantially exceeding chance. The antitype status of “Isolated Anchoring” is particularly informative—only 8.6% of rulings exhibited anchoring without framing, compared to an expected 19.2%, suggesting that the normative anchor’s analytical pathway almost inevitably activates its embedded qualitative frame.

At the system level, the “Full Hierarchical Transmission” configuration (10.9% observed vs. 6.1% expected) was directionally over-represented, though its marginal status after Holm correction means that the inference that transmission extends reliably to the second tier—linking framing to availability—requires confirmation in larger samples. The “Independent Reasoning” configuration’s over-representation (34.4% vs. 21.1% expected) provides a counterbalance: a substantial minority of post-Minutes rulings exhibited none of the three coded biases, indicating that normative anchor capture is far from universal. This heterogeneity is consistent with Teichman et al.’s (2023) finding that bias susceptibility varies across individuals within the same professional group and suggests that individual differences in judges’ analytical independence moderate the normative anchor’s downstream transmission effects (Table 3).

**Table 3:** Significant bias combination patterns from Configural Frequency Analysis (Post-2019 Chinese VAM, *n* = 128).

Configuration	A	F	V	Observed	Expected	*z*	*p* (Holm)	Sample %	Predominant outcome	Theoretical label	Status
Anchor–frame coupling	1	1	0	29	17.89	2.63	0.009**	22.7%	Claim dismissed or partial support	Anchor–Frame Coupling	Type (↑)
Full transmission	1	1	1	14	7.85	2.20	0.028* (marginal after Holm)	10.9%	Mixed; high partial support	Full Hierarchical Transmission	Type (↑, marginal)
Independent reasoning	0	0	0	44	26.93	3.29	0.001**	34.4%	Evenly distributed	Independent Reasoning	Type (↑)
Isolated anchoring	1	0	0	11	24.52	−2.73	0.006**	8.6%	—	—	Antitype (↓)
Isolated framing	0	1	0	5	19.65	−3.31	0.001**	3.9%	—	—	Antitype (↓)
A1 F0 V1	1	0	1	7	10.75	−1.14	n.s.	—	—	—	Not significant
A0 F1 V1	0	1	1	6	8.61	−0.89	n.s.	—	—	—	Not significant
A0 F0 V1	0	0	1	12	11.80	0.06	n.s.	—	—	—	Not significant

A = Anchoring; F = Framing; V = Availability. Baseline: P(A) = 0.477, P(F) = 0.422, P(V) = 0.305. Expected frequencies under independence. *p < 0.05; **p < 0.01 (Holm-corrected). Marginal frequencies verified: A+ = 29 + 14 + 7 + 11 = 61; F+ = 29 + 14 + 6 + 5 = 54; V+ = 14 + 7 + 6 + 12 = 39; Total = 128.

#### 4.2.1 Exploratory cross-jurisdictional comparison (H3)

The cross-jurisdictional comparison was limited to framing and availability, as the coding criteria for anchoring cannot be equivalently applied to American rulings. Framing: Chinese 42.2% (54/128) vs. American 27.0% (17/63), χ^2^(1) = 3.94, *p* = 0.047, Cramér’s *V* = 0.144. Availability: Chinese 30.5% (39/128) vs. American 23.8% (15/63), χ^2^(1) = 0.87, *p* = 0.351, Cramér’s *V* = 0.067. These comparisons must be understood in light of a structural feature of the two datasets: the mean reasoning section length was 847 characters (SD = 412) for Chinese rulings, compared to 2,341 words (SD = 1,187) for American rulings. Longer reasoning mechanically increases the opportunity for alternative characterizations and fact-matching analyses to appear in the text, and since both coding criteria depend on the documented absence of such elements, the length disparity serves as a competing explanation that aligns with the institutional anchor hypothesis—depressing American coding rates regardless of any underlying cognitive differences. The framing difference (*V* = 0.144) is therefore best treated as a directional signal whose magnitude cannot be cleanly attributed to the presence or absence of an institutional anchor. This confound—alongside differences in legal doctrine, judicial hierarchy, judgment-writing conventions, and publication practices described in the Institutional Background—is one of the principal reasons H3 was designated exploratory from the outset, and why the American data are treated as an exploratory contrast rather than a causal benchmark (Table 4).

**Table 4:** Cross-jurisdictional comparison: Chinese VAM (post-2019) vs. American earn-out.

Bias	Chinese VAM (*n* = 128)	American earn-out (*n* = 63)	χ^2^(1)	*p*	Cramér’s *V*	Interpretation
Anchoring effect	61 (47.7%)	N/A	—	—	—	Coding criteria reference the Minutes; not applicable to U.S. rulings
Framing effect	54 (42.2%)	17 (27.0%)	3.94	0.047*	0.144	Significant but small; directionally consistent with H3
Availability heuristic	39 (30.5%)	15 (23.8%)	0.87	0.351	0.067	Not significant

Judicial reasoning detail	Chinese “Court’s opinion”	American “Court’s analysis”
Mean word count (SD)	847 characters (412)	2,341 words (1,187)
Implication for coding	Shorter reasoning → fewer observable alternative analyses → potentially higher bias coding	Longer reasoning → more alternatives discussed → potentially lower bias coding

*p < 0.05. Cramér’s *V* benchmarks: 0.10 = small, 0.30 = medium, 0.50 = large. Word count uses characters for Chinese and words for English; direct comparison is descriptive.

### 4.3 Robustness checks

Excluding Supreme People’s Court rulings (*n* = 11): H1 anchoring rate 46.2% (54/117), with the difference from the baseline remaining significant, χ^2^(1) = 5.12, *p* = 0.024, *V* = 0.180. H2 framing level change *β*₂ = 11.8 pp. (*p* = 0.031), availability trend change β₃ = 2.9 pp./year (*p* = 0.046)—direction and significance consistent with full-sample results.

*Post hoc* power analysis indicated that the H1 chi-square test (*n* = 170, *V* = 0.186, *α* = 0.05) achieved a power of 0.72, marginally below the conventional 0.80 threshold. This shortfall reflects the population constraint described in the Methodology rather than a sampling decision: the post-2019 cases meeting both inclusion criteria were finite at the time of collection, and enlarging the sample would have required either relaxing those criteria—at the cost of including rulings in which the Minutes were not substantively engaged—or extending the collection window into years not yet available. The H1 finding should accordingly be read as preliminary evidence of the ambiguity-amplification effect, with the expectation that the steadily growing body of published VAM rulings will permit higher-powered replication in the near term. ITSA estimation faced a similar constraint: 13 annual observation windows are an inherent feature of the intervention’s timing within the observation period, and, as noted in the Statistical Analysis Plan, the segmented regression results are interpreted as preliminary archival evidence rather than definitive causal estimates.

Cross-validation with Lin’s (2020) dataset on 34 temporally overlapping cases revealed an outcome classification concordance rate of 82.4% (28/34).

## 5 Discussion

### 5.1 Empirical support for the debiasing paradox

All three research questions received directionally consistent responses. H1 was supported: under the same policy instrument, anchoring in VAM rulings (47.7%) significantly exceeded private lending (26.2%), *V* = 0.186—at the low-to-mid end of Bystranowski et al.’s (2021) meta-analytic range, though the conservative three-element coding standard likely underestimates actual cognitive anchoring. H2 was supported, with framing showing an immediate level jump (*β*₂ = 12.4, *p* = 0.024) and availability showing a delayed trend increase (*β*₃ = 3.2, *p* = 0.027). H3’s exploratory comparison revealed a significant framing difference (V = 0.144), directionally consistent though modest.

The core significance lies not in demonstrating that anchoring exists—an established finding—but in providing evidence consistent with a normative anchor producing indirect effects through hierarchical transmission. Bennett (2014) discovered a direct anchoring effect of a numerical anchor in sentencing but did not examine whether that anchor triggered other cognitive biases. The present ITSA results yield a temporal pattern consistent with a biasing influence propagating along an “anchoring → framing → availability” chain. This finding extends Bennett’s analysis in two respects. First, it indicates that the anchoring mechanism operates not only through numerical displacement (judges sentencing closer to the guideline range) but also through qualitative channeling (judges adopting the analytical frame embedded in the prescriptive pathway). Second, it suggests that a single institutional anchor may generate cascading cognitive effects across multiple bias dimensions—a phenomenon that single-bias experimental paradigms cannot detect because they typically isolate one heuristic at a time.

Configural frequency analysis results converge with the ITSA findings at the configural level: the co-occurrence of anchoring and framing is a significant type (*z* = 2.63, corrected *p* = 0.009), each bias’s isolated configuration is a significant antitype, and the coupling pattern cannot be explained by a model in which the three biases operate independently. The antitype status of “Isolated Anchoring” carries particular theoretical weight—it indicates that anchoring without downstream framing is statistically rarer than chance would predict, implying that the normative anchor’s analytical pathway almost inevitably activates its embedded frame. The “Full Transmission” configuration was over-represented at the individual test level (*z* = 2.20, *p* = 0.028) but reached only marginal significance after Holm correction—suggesting that hierarchical transmission may extend to the second tier in some cases, though this inference is limited by the current sample size. The observed coupling requires a structural linkage of the kind proposed by the hierarchical transmission model.

The paradox thus receives support beyond the near-tautological conclusion that “anchoring increased after the Minutes.” The pattern is consistent with the Minutes having operated not only as a cognitive anchor but also, through the qualitative frame embedded in their pathway and the accumulation of Minutes-compliant precedents, as an influence on judges’ frame selection and precedent retrieval. Ly et al. (2023), documenting anchoring by prior information in emergency medical decision-making, reported an indirect effect pathway structurally similar to the transmission pattern recorded here.

Alternative explanations warrant acknowledgment. The post-Minutes rise in framing and availability may partly reflect changes in the intrinsic complexity of the VAM domain around 2019. Specifically, the period surrounding 2019 saw a surge in PE/VC exit-related disputes driven by macroeconomic tightening and fundraising cycle compression, which may have increased the proportion of factually complex cases entering the court system—cases in which judges, facing greater analytical difficulty, may have been independently more prone to framing shortcuts regardless of the Minutes’ influence. Additionally, the growing volume of VAM litigation may have expanded available precedents through a purely mechanical channel unrelated to cognitive transmission. The ITSA design controls for pre-intervention trends but cannot fully exclude other institutional variables coinciding with the Minutes’ issuance. The observation window extends only to 2024; whether hierarchical transmission effects will plateau or continue accelerating requires future tracking.

### 5.2 Institutional moderation and methodological reflections

Several structural features of the Chinese judicial system—case clearance evaluations, de facto binding higher court guidance, and peer scrutiny from mandatory judgment publication—collectively encourage reduced investment in independent analysis. Within this environment, invoking the Minutes’ pathway serves as both a cognitive shortcut and an institutional “safe strategy”: rulings following the Minutes’ pathway are less likely to be reversed on appeal or questioned in peer review, creating an institutional incentive structure that reinforces cognitive anchoring. Liu and Li’s (2019) finding that Chinese judges rationalize bias-affected decisions through doctrinal reasoning suggests the Minutes may function simultaneously as an anchor and a rationalization tool: judges can present what is essentially an anchoring-driven analytical pathway as a faithful application of an authoritative document. Through the lens of resource rationality (Lieder and Griffiths, 2020), accepting the normative anchor represents adaptive behavior under bounded cognitive resources—rational at the institutional incentive level, though constituting distortion at the cognitive bias level.

The American reference group provides a baseline for bias occurrence under “no institutional anchor” conditions. The framing effect coding rate in the American sample (27.0%), while lower than in the Chinese post-2019 sample (42.2%), is far from zero—indicating that framing occurs at a non-trivial frequency even in the absence of an institutional anchor. This suggests that framing in judicial reasoning arises from multiple sources: institutional anchors amplify it, but judges’ individual doctrinal preferences and the spontaneous framing effects of case facts also contribute independently. Liu et al.’s (2021) finding that Chinese judges are cognitively influenced by prior decisions carries explanatory weight here: even in a system that formally disclaims precedential constraint, de facto precedent effects operate through cognitive availability.

The core methodological limitation—“judgment text ≠ cognitive process”—warrants direct engagement. Process coding identifies textual reasoning patterns, not cognitive states. The absence of alternative reasoning or fact-matching argumentation in a written opinion allows for explanations other than cognitive bias. Drafting conventions favoring economical exposition, judicial economy under heavy caseloads, institutional norms that standardize opinion structure, and individual stylistic preferences could each produce textual patterns resembling those coded here. The conjunctive coding structure was adopted precisely with these alternatives in mind: an economical drafting style will typically account for the absence of one element, but a ruling is coded as bias-indicative only when all prescribed elements co-occur, which raises the evidentiary bar against mistaking brevity for bias. Reasoning section word count was likewise included as a control variable to address the concern that shorter opinions mechanically yield fewer documented alternatives, and the 82.4% outcome classification concordance with Lin's (2020) independently constructed dataset offers at least indirect assurance that the underlying case readings are not idiosyncratic. These safeguards narrow the inferential gap between text and cognition without closing it. Misclassification occurs in both directions: false positives (independent analysis conducted but alternatives not documented in the text) and false negatives (bias concealed through careful drafting—a possibility Liu and Li (2019) demonstrated is not merely hypothetical). This limitation affects the three hypotheses to different degrees. For H2 (ITSA), the limitation is partially mitigated: even if process coding carries a systematic false positive rate, that rate should remain roughly constant before and after the Minutes, since judgment-writing conventions did not shift abruptly in November 2019; pre-post changes in framing and availability therefore reflect genuine changes in the coded reasoning patterns rather than systematic coding noise. For H1 (cross-sectional comparison), this mitigation does not apply—judgment-writing conventions themselves differ between VAM and private lending rulings, the former involving more complex legal relationships and more numerous contested issues, where judges may omit alternative analytical pathways for reasons of length rather than cognitive anchoring; the resulting false positive rate may not be constant across the two case types, and some portion of the 47.7% versus 26.2% gap may reflect differences in drafting style rather than in anchoring susceptibility. For H3 (cross-jurisdictional comparison), the word count disparity already reported in the Exploratory Cross-Jurisdictional Comparison section (847 characters vs. 2,341 words) means that American rulings provide coders with substantially more observable reasoning, systematically reducing the probability that American samples trigger the coding criteria—a difference constituting a competing explanation for the H3 results and one of the reasons that hypothesis was designated exploratory.

An obvious way to validate coding inferences would be to ask judges themselves through interviews keyed to the coded rulings. This option is foreclosed in the present setting. Chinese judges are bound by deliberation confidentiality rules that prevent them from discussing the reasoning of specific adjudicated cases with researchers. Even where retrospective interviews were possible, accounts of one’s own past reasoning are subject to the same *post hoc* rationalization that Liu and Li (2019) documented in written opinions, with recall error and social desirability added on top. Interview validation would thus substitute one layer of reconstructed reasoning for another rather than reaching the underlying cognition. The findings reported here are best read as evidence about the distribution of anchor-consistent reasoning patterns in published judgments. What links these patterns to the proposed cognitive mechanism is not any single coding decision but the convergence of three independent strands: the conjunctive criteria, the distinct temporal signatures predicted and then observed in the ITSA, and the configural coupling identified by the CFA. Direct validation against cognitive measures remains a task for future work using vignette experiments and think-aloud protocols, as outlined in the Conclusion.

Berthet’s (2022) cross-occupational findings, along with Saposnik et al.’s (2016) clinical guideline anchoring paradox, suggest that the debiasing paradox represents a general institutional tension—any arrangement unifying professional judgment through standardized guidance risks producing cognitive biases while reducing surface-level inconsistency. This tension is not unique to the Chinese judicial system but is inherent in the structural contradiction between standardization and professional judgment.

#### 5.2.1 Debiasing implications

The recommendations developed below are theoretically grounded propositions derived from the present findings and the debiasing literature; they have not themselves been empirically tested, and each is paired in the Conclusion with a design through which future research can evaluate it. Soll et al. (2015) distinguished two pathways: “changing the person” (training) and “changing the environment” (institutional design). Given that Korteling et al.’s (2021) systematic review found limited transfer of debiasing training and Scopelliti et al. (2015) demonstrated that professionals systematically underestimate their own bias susceptibility, reliance on individual awareness alone is insufficient. Three institutional design recommendations follow.

Addressing anchoring: Introducing a “Minutes applicability reasoning” requirement—judges invoking the Minutes must explicitly demonstrate the match between case-specific facts and the Minutes’ stipulated conditions. This structurally reduces the incidence of Element B (absent fact-matching) in the anchoring coding criterion.

Addressing framing: Designing a “dual-framework reasoning” procedure—judges must analyze repurchase clauses separately from contract law and company law perspectives before selecting a characterization. Mussweiler et al.’s (2000) “consider-the-opposite” strategy attenuates anchoring; mandating an alternative framework disrupts single-frame monopoly. Aharoni et al. (2022), in Frontiers in Psychology, confirmed that altering informational frames substantively changes judicial sentencing decisions.

Addressing availability: Establishing systematic feedback—periodic statistical distribution reports on VAM adjudicatory outcomes, replacing availability-driven precedent retrieval with base-rate information. Morewedge et al. (2015) demonstrated medium-to-large debiasing effect sizes persisting for eight weeks, and Sellier et al. (2019) showed field transfer of such training effects.

## 6 Conclusion

This study examined the debiasing paradox—whether the Ninth Meeting Minutes, designed to unify adjudicatory standards, produced new systematic biases through hierarchical transmission of a normative anchor—using Chinese VAM adjudication as the empirical setting. Based on process coding of 217 Chinese VAM rulings, 42 private lending rulings, and 63 American earn-out rulings, the following findings emerged, which should be considered preliminary archival evidence given the design constraints discussed below. Under the same policy instrument, anchoring was significantly stronger in VAM rulings than in private lending rulings (47.7% vs. 26.2%, V = 0.186), indicating that legal ambiguity amplifies normative anchor effects. Following the Minutes, framing exhibited an immediate level jump (*β*₂ = 12.4, *p* = 0.024), while availability showed a delayed trend increase (β₃ = 3.2, *p* = 0.027), corresponding to downstream triggering and indirect reinforcement, consistent with the hierarchical transmission hypothesis. CFA cross-validated the anchoring-framing transmission relationship through significant over-representation of “Anchor–Frame Coupling” (z = 2.63, *p* = 0.009) and antitype status of isolated configurations; the directional over-representation of the “Full Transmission” configuration suggests a possible three-tier chain, though confirmation in larger samples is needed. Exploratory cross-jurisdictional comparison showed higher framing frequency in Chinese VAM rulings, which is directionally consistent with the institutional anchor hypothesis.

Theoretically, this study proposed and tested the debiasing paradox and normative anchor hierarchical transmission, extending the anchoring typology to qualitative institutional anchors with downstream transmission capacity. Methodologically, process coding and the differentiated cross-sectional/ITSA strategy offer a replicable framework for archival cognitive bias research.

Several limitations constrain these conclusions. The coding relies on textual inference rather than direct cognitive observation: the criteria identify reasoning patterns consistent with anchoring, framing, and availability, while competing explanations rooted in drafting conventions and judicial economy, although partially accounted for by the conjunctive coding structure and word-count controls, cannot be fully excluded—particularly for the cross-sectional H1 comparison, where writing conventions themselves differ between VAM and private lending rulings. Interview-based validation of the coding inferences was not available for decided cases. The H1 test achieved a power of 0.72, below the conventional threshold, a constraint imposed by the population of qualifying rulings rather than by sampling choices; the ITSA estimates rely on 13 annual windows and therefore carry the instability of small-sample time series models. The American reference data are subject to the reasoning-length confound documented above, which operates in the same direction as the institutional anchor hypothesis and restricts H3 to a directional reading. Finally, the case-level design cannot capture within-judge variation in bias susceptibility, leaving the individual-difference moderation suggested by the CFA heterogeneity for future work. These limitations collectively advise reading the findings as preliminary archival evidence for the debiasing paradox, to be consolidated through the replication and experimental validation pathways outlined below.

Future research can extend this work along three fronts. The validity of process coding itself can be tested with direct cognitive measures: vignette experiments manipulating the presence of a normative anchor would establish whether the textual patterns coded here track the underlying adjustment deficit, and think-aloud protocols with judges reasoning through hypothetical VAM disputes—free of the deliberation confidentiality constraints attached to decided cases—would allow checks of coding inferences against contemporaneous verbal reports. The three institutional recommendations advanced in the Discussion are each open to empirical evaluation: an applicability reasoning requirement can be tested experimentally by manipulating whether participants must document fact-matching before invoking the Minutes and observing the effect on Element B incidence; the dual-framework procedure can be evaluated in a controlled comparison of single-framework and mandated dual-characterization conditions, building on the consider-the-opposite paradigm (Mussweiler et al., 2000) and the frame-alteration evidence of Aharoni et al. (2022); and should any court adopt periodic outcome-distribution reporting, the resulting natural experiment could be assessed with the same interrupted time series design employed here. Finally, the accumulating body of post-2024 VAM rulings will permit higher-powered replication over a longer post-intervention window, clarifying whether the hierarchical transmission effects documented here plateau or continue to build, and whether the debiasing paradox generalizes to other domains governed by standardized guidance instruments.

## Data Availability

The raw data supporting the conclusions of this article will be made available by the authors, without undue reservation.

## References

[ref1] Aharoni E. Kleider-Offutt H. M. Brosnan S. F. Hoffman M. B. (2022). Nudges for judges: an experiment on the effect of making sentencing costs explicit. Front. Psychol. 13:889933. doi: 10.3389/fpsyg.2022.889933, 35712212 PMC9197476

[ref2] Bennett M. W. (2014). Confronting cognitive “anchoring effect” and “blind spot” biases in federal sentencing: a modest solution for reforming a fundamental flaw. J. Crim. Law Criminol. 104, 489–534.

[ref3] Bernal J. L. Cummins S. Gasparrini A. (2017). Interrupted time series regression for the evaluation of public health interventions: a tutorial. Int. J. Epidemiol. 46, 348–355. doi: 10.1093/ije/dyw098 27283160 PMC5407170

[ref4] Berthet V. (2022). The impact of cognitive biases on professionals’ decision-making: a review of four occupational areas. Front. Psychol. 12:802439. doi: 10.3389/fpsyg.2021.802439, 35058862 PMC8763848

[ref5] Bystranowski P. Janik B. Próchnicki M. Skórska P. (2021). Anchoring effect in legal decision-making: a meta-analysis. Law Hum. Behav. 45, 1–23. doi: 10.1037/lhb0000438, 33734746

[ref6] Englich B. Mussweiler T. Strack F. (2006). Playing dice with criminal sentences: the influence of irrelevant anchors on experts’ judicial decision making. Personal. Soc. Psychol. Bull. 32, 188–200. doi: 10.1177/0146167205282152, 16382081

[ref7] Enough B. Mussweiler T. (2001). Sentencing under uncertainty: anchoring effects in the courtroom. J. Appl. Soc. Psychol. 31, 1535–1551. doi: 10.1111/j.1559-1816.2001.tb02687.x

[ref8] Glöckner A. Englich B. (2015). When relevance matters: anchoring effects can be larger for relevant than for irrelevant anchors. Soc. Psychol. 46, 4–12. doi: 10.1027/1864-9335/a000214

[ref9] Guthrie C. Rachlinski J. J. Wistrich A. J. (2001). Inside the judicial mind. Cornell Law Rev. 86, 777–830.

[ref10] Guthrie C. Rachlinski J. J. Wistrich A. J. (2007). Blinking on the bench: how judges decide cases. Cornell Law Rev. 93, 1–44.

[ref11] Kahneman D. (2011). Thinking, Fast and Slow. New York, NY: Farrar, Straus and Giroux.

[ref12] Kahneman D. Klein G. (2009). Conditions for intuitive expertise: a failure to disagree. Am. Psychol. 64, 515–526. doi: 10.1037/a0016755, 19739881

[ref13] Korteling J. E. Gerritsma J. Y. J. Toet A. (2021). Retention and transfer of cognitive bias mitigation interventions: a systematic literature study. Front. Psychol. 12:629354. doi: 10.3389/fpsyg.2021.629354, 34456780 PMC8397507

[ref14] Krippendorff K. (2018). Content Analysis: An Introduction to its Methodology. 4th Edn Thousand Oaks, CA: SAGE.

[ref15] Lieder F. Griffiths T. L. (2020). Resource-rational analysis: understanding human cognition as the optimal use of limited computational resources. Behav. Brain Sci. 43:e1. doi: 10.1017/S0140525X1900061X, 30714890

[ref16] Lin L. (2020). Contractual innovation in China’s venture capital market. Eur. Bus. Organ. Law Rev. 21, 101–138. doi: 10.1007/s40804-020-00184-x

[ref17] Linden A. (2015). Conducting interrupted time-series analysis for single- and multiple-group comparisons. Stata J. 15, 480–500. doi: 10.1177/1536867X1501500208

[ref18] Linden A. (2017). A comprehensive set of postestimation measures to enrich interrupted time-series analysis. Stata J. 17, 73–88. doi: 10.1177/1536867X1701700105

[ref19] Liu J. Z. Klöhn L. Spamann H. (2021). Precedents and Chinese judges: an experiment. Am. J. Comp. Law 69, 93–135. doi: 10.1093/ajcl/avab009

[ref20] Liu J. Z. Li X. (2019). Legal techniques for rationalizing biased judicial decisions: evidence from experiments with real judges. J. Empir. Legal Stud. 16, 630–670. doi: 10.1111/jels.12229

[ref21] Ly D. P. Shekelle P. G. Song Z. (2023). Evidence for anchoring bias during physician decision-making. JAMA Intern. Med. 183, 818–823. doi: 10.1001/jamainternmed.2023.2366, 37358843 PMC10294014

[ref22] McHugh M. L. (2012). Interrater reliability: the kappa statistic. Biochem. Med. 22, 276–282. doi: 10.11613/BM.2012.031, 23092060 PMC3900052

[ref23] Morewedge C. K. Yoon H. Scopelliti I. Symborski C. W. Korris J. H. Kassam K. S. (2015). Debiasing decisions: improved decision making with a single training intervention. Policy Insights Behav. Brain Sci. 2, 129–140. doi: 10.1177/2372732215600886

[ref24] Mussweiler T. Strack F. Pfeiffer T. (2000). Overcoming the inevitable anchoring effect: considering the opposite compensates for selective accessibility. Personal. Soc. Psychol. Bull. 26, 1142–1150. doi: 10.1177/01461672002611010

[ref25] Olaborede A. Meintjes-Van der Walt L. (2020). Cognitive bias affecting decision-making in the legal process. Obiter 41, 806–830. doi: 10.17159/obiter.v41i4.10489

[ref26] Page M. J. McKenzie J. E. Bossuyt P. M. Boutron I. Hoffmann T. C. Mulrow C. D. . (2021). The PRISMA 2020 statement: an updated guideline for reporting systematic reviews. BMJ 372:n71. doi: 10.1136/bmj.n71, 33782057 PMC8005924

[ref27] Rachlinski J. J. Wistrich A. J. (2017). Judging the judiciary by the numbers: empirical research on judges. Annu. Rev. Law Soc. Sci. 13, 203–229. doi: 10.1146/annurev-lawsocsci-110615-085032

[ref28] Rachlinski J. J. Wistrich A. J. (2018). Gains, losses, and judges: framing and the judiciary. Notre Dame Law Rev. 94, 521–582.

[ref29] Saposnik G. Redelmeier D. Ruff C. C. Tobler P. N. (2016). Cognitive biases associated with medical decisions: a systematic review. BMC Med. Inform. Decis. Mak. 16:138. doi: 10.1186/s12911-016-0377-1, 27809908 PMC5093937

[ref30] Scopelliti I. Morewedge C. K. McCormick E. Min H. L. Lebrecht S. Kassam K. S. (2015). Bias blind spot: structure, measurement, and consequences. Manag. Sci. 61, 2468–2486. doi: 10.1287/mnsc.2014.2096, 19642375

[ref31] Sellier A.-L. Scopelliti I. Morewedge C. K. (2019). Debiasing training improves decision making in the field. Psychol. Sci. 30, 1371–1379. doi: 10.1177/0956797619861429, 31347444

[ref32] Soll J. B. Milkman K. L. Payne J. W. (2015). “A user’s guide to debiasing,” in The Wiley Blackwell Handbook of Judgment and Decision Making, Vol. II, eds. Keren G. Wu G. (Chichester: Wiley-Blackwell), 924–951.

[ref33] Spamann H. Klöhn L. (2016). Justice is less blind, and less legalistic, than we thought: evidence from an experiment with real judges. J. Leg. Stud. 45, 255–280. doi: 10.1086/688861

[ref34] Spamann H. Klöhn L. Jamin C. Khanna V. Liu J. Z. Mamidi P. . (2021). Judges in the lab: no precedent effects, no common/civil law differences. J. Leg. Anal. 13, 110–126. doi: 10.1093/jla/laab001

[ref35] Teichman D. Zamir E. Ritov I. (2023). Biases in legal decision-making: comparing prosecutors, defense attorneys, law students, and laypersons. J. Empir. Legal Stud. 20, 852–894. doi: 10.1111/jels.12365

[ref36] Tversky A. Kahneman D. (1973). Availability: a heuristic for judging frequency and probability. Cogn. Psychol. 5, 207–232. doi: 10.1016/0010-0285(73)90033-9

[ref37] Tversky A. Kahneman D. (1974). Judgment under uncertainty: heuristics and biases. Science 185, 1124–1131. doi: 10.1126/science.185.4157.1124, 17835457

[ref38] Tversky A. Kahneman D. (1981). The framing of decisions and the psychology of choice. Science 211, 453–458. doi: 10.1126/science.7455683, 7455683

[ref39] von Eye A. Mair P. Mun E.-Y. (2010). Advances in Configural Frequency Analysis. New York, NY: Guilford Press.

[ref40] von Eye A. Wiedermann W. (2022). Configural Frequency Analysis: Foundations, Models, and Applications. Berlin; Heidelberg: Springer.

[ref41] Wang J. (2022). Valuation adjustment mechanism in China: a risk management strategy or risk-triggering device? Hong Kong Law J. 52, 763–778.

